# The interlocking process in molecular machines explained by a combined approach: the nudged elastic band method and machine learning potential

**DOI:** 10.1039/d5sc08303f

**Published:** 2026-01-13

**Authors:** Lucio Peña-Zarate, Alberto Vela, Jorge Tiburcio

**Affiliations:** a Department of Chemistry, Centro de Investigación y de Estudios Avanzados (Cinvestav) Avenida IPN 2508 Mexico City 07360 Mexico avela@cinvestav.mx jtiburcio@cinvestav.mx

## Abstract

Engineering molecular machines requires a precise knowledge of the mechanisms involved in programmed motions. Among artificial molecular machines, rotaxanes have emerged as a prominent model due to their ability to perform diverse and controlled motions, such as threading, shuttling, and pirouetting. In this work, we present a reliable theoretical framework to describe the threading motion during the assembly of rotaxane-like complexes. Our approach combines the climbing image nudged elastic band method with the ANI-1ccx neural network potential, trained with gold-standard data. Energetic and structural variations along a normalized displacement coordinate allowed an accurate atomistic description of the threading process of different dumbbell-shaped molecules (axles) through the cavity of two different macrocyclic hosts (tori). Using the methodology proposed herein, two key steps are identified: stabilization through hydrogen bonds, which we call the claw mechanism, and the expansion of the macrocycle. An energy decomposition analysis, performed by single-point calculations on selected structures, allows an analysis of the role of steric and electrostatic effects in the structural stabilization of supramolecular assemblies. We find that, although ANI-1ccx was not explicitly trained for charged systems, this neural network potential effectively discriminates among various charged states. Furthermore, calculated potential energy barriers are in good agreement with reported experimental free energy barriers. The featured methodology has the potential to become a fundamental artificial-intelligence-based tool for the study of diverse motions observed in supramolecular systems.

## Abstract 2

Engineering molecular machines requires a precise knowledge of the mechanisms involved in programmed motions.

## Graphical abstract

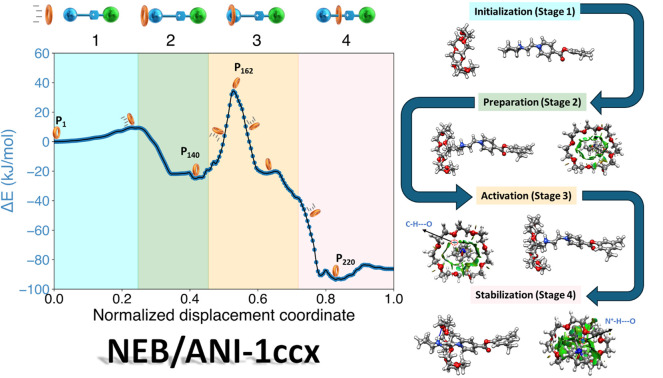

## Introduction

Artificial molecular machines are synthetic systems designed to mimic the movement of macroscopic machines by responding to an external stimulus. These chemical systems share several similarities with macroscopic machines, such as the need for an energy input for operation, the relative movements of their components, the ability to monitor and control their functions, the capacity to repeat operation cycles, the time required to complete a full cycle, and the purpose of their operation.^1,2^

Mechanically interlocked molecules (MIMs), like rotaxanes and catenanes, have emerged as prototypical artificial molecular machines because of their ability to perform large-amplitude motions in response to an energy source, while maintaining structural integrity.^3^ Rotaxanes, in particular, are a family of supramolecular host–guest complexes consisting of a dumbbell-shaped guest (axle) encircled by one or more macrocyclic hosts (tori), with both ends of the guest extending outside the ring.^4^ A typical method to assemble rotaxanes involves threading the torus over one of the terminal groups on the axle, a process that is influenced by flexibility, size-complementarity, and steric and electrostatic effects between the components.^5^

Given the importance of MIMs, it is essential to understand the energetic profile underlying their dynamics through an atomistic model. The desired model must reveal how different non-covalent interactions and conformational changes interplay along the path of molecular motion. Additionally, estimating energy barriers could reveal how chemical modifications in the host and guest influence the assembly process. This method should also provide the molecular basis for rationalizing experimental data.

Computational tools have played a crucial role in understanding the mechanisms of shuttling, dissociation, aggregation, and conformational changes in supramolecular systems. These include methods such as molecular dynamics,^6–10^ metadynamics,^11–14^ nudged elastic band (NEB),^15,16^ and calculations of electronic structure.^17–21^ However, these techniques face limitations due to the complexity of supramolecular systems, including their size and dynamic processes,^22^ as well as the challenge of selecting an appropriate collective variable^23–25^ that can describe a coherent reaction coordinate for the process. As a result, it remains challenging for computational studies to quickly and accurately identify these mechanisms.^22^

One approach to addressing this challenge involves utilizing atomic neural network potentials (NNPs) as an alternative to conventional electronic structure approaches.^26^ The majority of these potentials are developed based on datasets derived from DFT calculations. An illustrative example is the potential employing the Polarizable Atom Interaction Neural Network (PaiNN), which has been utilized in transition-state searches in conjunction with the NEB method.^27^ This helps to obtain insights into the reaction mechanisms of various reactions, at speeds approximately three orders of magnitude faster than NEB/DFT calculations, demonstrating the capacity of PaiNN to expedite transition-state search techniques with NEB. The combination of NEB with reliable electronic structure calculations for transition-state analysis is markedly more computationally intensive than pairing NEB with a well-trained neural network.^28^

Another set of neural network potentials is the ANAKIN-ME family, which has been utilized in a variety of applications, including high-precision calculations of thermochemical reaction values,^29–32^ determination of conformational change,^33–37^ molecular dynamics simulations,^38–40^ structural searches for new drugs,^41^ and the prediction of absorption, distribution, metabolism, and excretion (ADME).^42^ Among the ANAKIN-ME family, particular attention is given to ANI-1ccx, which was trained using the gold standard (CCSD(T)*/CBS) of molecular electronic structure theory. This model has produced reasonable predictions of the binding structures of ligands in relation to experimental data, even though electrically charged molecules are outside the scope of the ANI-1ccx training set.^38^ This last study exemplifies the ability of the ANI-1ccx neural network to deliver coherent responses to conformational analysis, considering that the ANI-1ccx version cannot account for the charge and multiplicity of the systems under consideration.

**Fig. 1:**
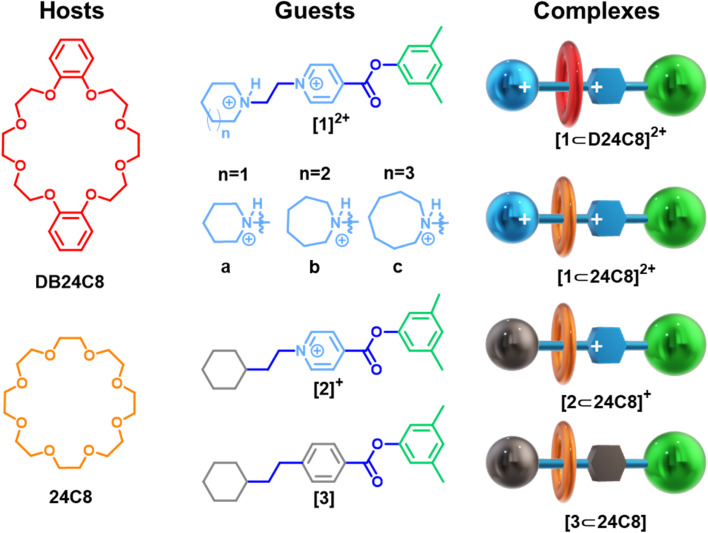
Chemical and cartoon representations of hosts, guests, and complexes. The notation used to designate the complexes is [Guest⊂Host] where Guest denotes the axle: 1 corresponds to doubly charged axles, 2 to singly charged axles, and 3 to neutral axles; *n* indicates the size of the cyclic ammonium end-group on the axle: *n* = 1 (six-membered, piperidinium), *n* = 2 (seven-membered, azepanium), and *n* = 3 (eight-membered, azocanium). Hosts, the tori, are 24-crown-8 ether (24C8) or dibenzo-24-crown-8 ether (DB24C8).

The study described herein utilizes the ANI-1ccx potential in conjunction with the NEB method to elucidate the assembly processes involved in the formation of the rotaxanes depicted in Fig. 1. These rotaxanes feature asymmetric, dumbbell-shaped axles, with a bulky ester substituent on one end, acting as a stopper for the two investigated 24-membered macrocycles: 24-crown-8 (24C8) and dibenzo-24-crown-8 (DB24C8). The presence of one stopper on the axle allows macrocycle threading only over the other terminus. Guest family [1]^2+^ is composed of positively doubly charged axles, all of which have a pyridinium group linked *via* an ethyl bridge to cyclic ammonium substituents of different sizes, varying from piperidinium [1a]^2+^, to azepanium [1b]^2+^, and azocanium [1c]^2+^. An experimental study on the stability and association rates of these axles with the aforementioned macrocycles was previously described in the literature by our group.^43,44^ To explore the effect of charge on the formation of the [1a]^2+^ complex, [2]^+^ and [3] axles with 24C8 were selected as model systems, in which nitrogen atoms in the axle were replaced with carbon atoms to produce both a singly charged and a neutral variant. The shape of the energy profile during threading is dictated by non-covalent interactions and by the combined flexibility of the macrocycle and the guest substituents, factors determining whether the profile is smooth or rough. Furthermore, as our results show, even though ANI-1ccx neither accepts charge and multiplicity as inputs nor was trained on charged systems, its architecture allows it to indirectly capture charge effects, reproducing stability trends across neutral, and singly or doubly charged rotaxanes.

The proposed NEB/ANI-1ccx methodology allowed us to obtain detailed energy profiles consisting of plots of the association energy as a function of the normalized displacement coordinate (NDC). This allows a description of the conformational changes occurring in the macrocycles and the axles, and in conjunction with single-point DFT calculations together with non-covalent interaction (NCI) analysis and Shubin Liu's energy decomposition analysis (EDA-SBL), reveals the most important interactions that intervene in the ring translation over and between different end groups, allowing better comprehension of the interlocking mechanisms. These points are addressed in the Results and discussion section. The energy barriers for the interlocking processes are obtained as differences in potential energies calculated with ANI-1cxx, and they are found to be in good agreement with experimental activation energies. Finally, we draw general conclusions about the mechanisms involved in the formation of rotaxanes, using an atomistic model, as well as the implementation of NEB/ANI-1ccx as an alternative strategy to address association and translation processes in supramolecular systems.

### Methodology

This section describes the methodological approach that combines the climbing image NEB with the NNP ANI-1ccx. The analysis tools used to characterize the interactions and energy contributions are explained in the SI.

### Nudged elastic band (NEB)

This is a double-ended transition-state search method that approximates the reaction path on the potential energy surface by interpolating an initial path between the reactant and product, discretized by a system replica set called images.^45^ With these images, an initial path is constructed, and subsequently, an optimization algorithm is used to iteratively relax a discrete path with the *N* images until convergence to an approximate minimum-energy path is achieved. The reaction coordinate is the normalized displacement coordinate, which is the accumulated distance obtained by adding the Euclidean distances between each consecutive pair of images, which is evaluated as 
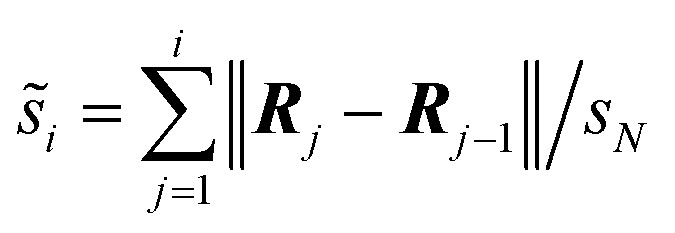
, for *i* = 1,2,…,*N*, where ***R***_*j*_ are the atomic coordinates of the *j*th image, *N* is the number of images, and *s*_*N*_ is the total length of the path. In this work, the normalized displacement coordinate (NDC) is used to build the energy profiles.

Several modifications to the NEB method exist; notably, the preconditioned chain method^46^ is designed to handle poorly conditioned potentials, such as those in structures with significant variations in rigidity, enabling faster convergence. However, it does not guarantee that the highest-energy structure, the transition state, can be found. The climbing image nudged elastic band method addresses this by allowing a specific image to behave differently, following variable spring constants and moving towards the highest-energy structure.^47^

#### ANI-1ccx

The ANI-1ccx potential was developed through transfer learning, initially utilizing DFT calculations obtained with ωB97X/6-31G* for systems composed of carbon, hydrogen, nitrogen, and oxygen, specifically from the ANI-1x dataset comprising five million out-of-equilibrium molecular conformations. Subsequently, it was retrained using a significantly smaller dataset: approximately 500 000 selected conformations from ANI-1x, using the CCSD(T)*/CBS level of accuracy, where the asterisk indicates that the domain-based local pair natural orbital approximation (DLPNO) was used.^48,49^ The resulting model operates at roughly nine orders of magnitude faster than DFT calculations. Through the transfer learning process, it not only refines the energy predictions but also improves the accuracy of force calculations. These achievements are enabled by extensive training and an advanced neural network architecture. The network consists of a series of hidden layers, with two of these layers being actively adjusted to optimize the transfer learning process, while the remaining two layers remain fixed to reduce the number of trainable parameters. This approach effectively prevents overfitting to the smaller CCSD(T)*/CBS dataset.^50^

#### NEB/ANI-1ccx

Our methodology yields high-resolution reaction coordinates, specifically the normalized displacement coordinate. This is because NEB constructs the reaction path directly in the full atomic configuration space through a chain of images representing complete geometries.^51^ Thus, unlike metadynamics, NEB does not require a reduction in dimensionality or the selection of collective variables, allowing an accurate description of the pathway and energy barriers without loss of information. This is achieved through the application of the climbing image NEB method, which is integrated *via* the Atomic Simulation Environment (ASE)^52^ into the ANI-1ccx neural network architecture and the LBFGS optimizer. The NDCs provide a thorough description of the assembly process in the rotaxane-type systems under investigation.

#### Shubin Liu's energy decomposition analysis (EDA-SBL)

This analysis reveals how steric, electrostatic, and quantum effects govern supramolecular assembly. In the EDA-SBL framework, the total energy is partitioned as:*E*_t_ = *E*_s_ + *E*_e_ + *E*_q_where *E*_s_ (steric energy) is associated with the Weizsäcker contribution to the kinetic energy functional, *E*_e_ (electrostatic energy) describes the direct interelectronic coulombic interaction, and *E*_q_ (quantum energy) represents the exchange–correlation and charge delocalization/transfer contributions.^53,54^

In this work, we present energy variations relative to an internal reference, defined as the P_1_ structure of each system. Thus, for any selected point ***P***_i_ along the energy profile, the reported values correspond to the differences with respect to P_1_, providing a clearer picture of the energetic contributions throughout the assembly process.

#### Computational details

All NEB calculations were performed by turning on the climbing image method and the string method^46^ in ASE. The computations utilized 125 images and linear interpolation, using the ANI-1ccx neural network potential trained from CCSD(T)*/CBS calculations data through TorchANI, a PyTorch-based program for ANI (ANAKIN-ME) deep-learning model training/inference. The Limited memory Broyden–Fletcher–Goldfarb–Shanno (LBFGS) optimization algorithm was used, with a maximum force value of max(|F|) < 0.1 eV Å^−1^, to achieve convergence of the calculations.

The reactant and product configurations used as input for the NEB calculations were aligned and optimized using the LBFGS method (ASE package), with the ANI-1ccx potential incorporated as an external calculator, using a convergence criterion of max(|F|) < 1 × 10^−4^ eV Å^−1^. Two NEB calculations were performed using three initial structures, with one serving as the product of the first NEB and the reactant of the second. To verify that the optimized structures of the reactant and product are local minima on the potential energy surface, a frequency analysis by finite differences was performed using ASE with ANI-1ccx as the calculator.

The structure used as input for the first NEB calculation, corresponding to the configuration with the axle and macrocycle with a very large separation, was also optimized. Its frequency analysis reveals the presence of imaginary modes that are the relative translational and rotational motions characteristic of supramolecular systems.^55^

For the NCI and EDA-SBL analyses, single-point DFT calculations were performed, using a selected set of images generated from the NEB/ANI-1ccx calculation trajectories. The exchange–correlation energy functional used was the non-empirical global hybrid with dispersion PBE0-D3, and the orbital basis def2TZVP.^56,57^ In all cases, SCF convergence was achieved, using the quadratically convergent SCF procedure (SCF = QC).^58^ All the DFT calculations were done using Gaussian 09.^59^ The NCI and EDA analyses were done using Multiwfn.^60^ For the NCI calculations, a 3D grid with 0.1 Å spacings along each Cartesian axis was used.

## Results and discussion

**Fig. 2:**
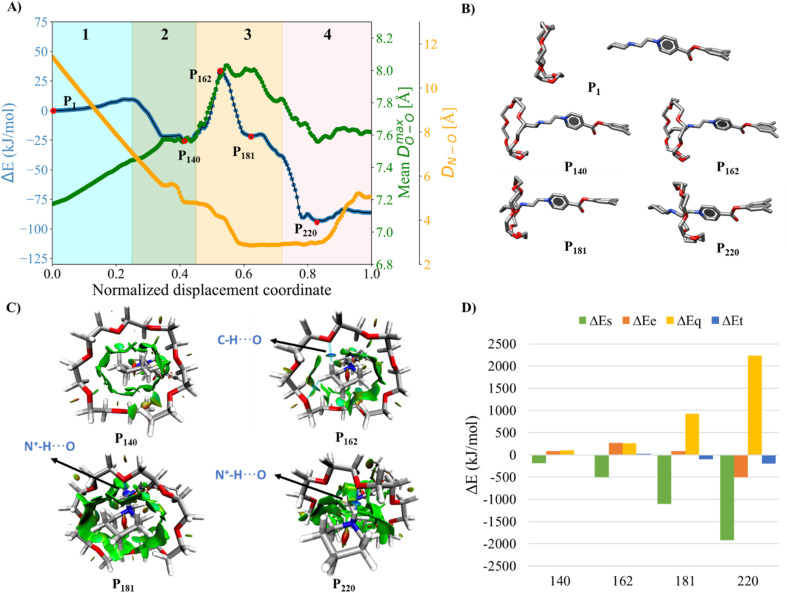
(A) Energy profile (blue curve), average maximum distance between oxygen atoms in 24C8 (*D*^max^_OO_, green curve), and donor–acceptor distance of the hydrogen bond (*D*_N⋯O_, orange curve) during the assembly process of [1a⊂24C8]^2+^. (B) Structures P_1_, P_140_, P_162_, P_181,_ and P_220_ corresponding to selected points along the curve. For clarity, hydrogen atoms are omitted. (C) Isosurfaces of the reduced density gradient 
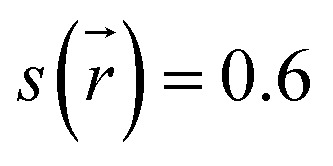
 colored according to sign(*λ*_2_) for the images P_140_, P_162_, P_181_ and P_220_. (D) EDA analysis for the corresponding images, showing the contributions to the energy, using P_1_ as reference.

Initially, we studied the threading process of the linear guest [1a]^2+^ through the cavity of the 24C8 macrocycle. The process is described by an NDC derived from successive images generated by NEB. Trajectories were built from 250 images from two different NEB calculations. The energy of each structure was calculated using the ANI-1ccx potential. A graphical representation of the energy profile during the threading along the NDC is shown in Fig. 2A. This figure also depicts the average maximum distance between pairs of oxygen atoms in the macrocycle *D*^max^_OO_ (green curve), and the hydrogen-bond donor–acceptor distance (orange curve). These two geometrical parameters provide useful information about the conformational changes involved in the intertwining process, as well as the formation and breaking of non-covalent interactions.

The assembly process can be divided into four stages, labeled and colored in Fig. 2A, based on the energy behavior along the NCD.

(1) Initialization (Stage 1). The components, axle and torus, interact weakly, and the energy undergoes very small changes; neither molecule exhibits significant geometric distortion.

(2) Preparation (Stage 2). The two molecular species interact attractively; energy drops, and axle and torus align to facilitate molecular assembly.

(3) Activation (Stage 3). The torus slides over the axle terminus, overcoming unfavorable steric interactions. This step determines the energy barrier for the assembly of the rotaxane complex and contains the transition state (see structure P_162_ in Fig. 2B).

(4) Stabilization (Stage 4). The torus reaches the positively charged region on the axle and encircles the ethylene group, providing the lowest energy conformation (see structure P_220_ in Fig. 2B); the macrocycle undergoes significant geometrical rearrangements, which are reflected in the parameter *D*^max^_OO_.

In the initialization stage, the torus (macrocycle) and the axle (guest) are nearly non-interacting species. This was confirmed by comparing the energy of the NEB initial structure with the sum of the energies of the isolated macrocycle and guest. In all studied systems, the interaction energy at this initial stage is zero. This is further supported by the NCI analysis, which shows no isosurfaces between the two species (see Fig. S7).

During preparation, the torus approaches the axle, resulting in structures that are more stable than the non-interacting species, with an energy decrease of about −25 kJ mol^−1^. This energy change is strongly linked to weak interactions caused by the proximity of the macrocycle to the guest, as can be seen by the NCI isosurfaces in the P_140_ complex (Fig. 2C), which are attributed to van der Waals interactions between host and guest.

In the activation stage, the 24C8 macrocycle slides over the cyclic ammonium located at the axle terminus, inducing significant conformational changes and cavity expansion. At the highest-energy point (P_162_ in Fig. 2C), a C–H⋯O hydrogen bond at 3.4 Å is observed. After overcoming an energy barrier of 59 kJ mol^−1^, the macrocycle approaches the nitrogen of the piperidinium group on the axle, resulting in the formation of an intermolecular hydrogen bond N^+^–H⋯O (orange curve) with a donor–acceptor distance of 2.8 Å. This set of non-covalent interactions stabilizes the complex formed during this activation stage. The EDA analysis indicates that in the P_162_ complex, the electrostatic contribution is unfavorable (Fig. 2D), as it has a positive value. This occurs because the macrocycle is still far from the region where the positive charge is mainly located. As the process continues, the macrocycle moves closer to the ammonium group, reducing the positive electrostatic energy in the P_181_ structure. Additionally, proximity to the ammonium group on the axle facilitates a strong hydrogen bond of the N^+^–H⋯O type between the macrocycle and the ammonium group, as shown by NCI analysis.

Finally, in the stabilization stage, the macrocycle reaches the minimum energy structure P_220_. The N^+^–H⋯O hydrogen bond displays slight variations in distance compared to that observed in P_181_. By maintaining this bond, the upper part of the macrocycle remains anchored to the axle, while the lower part shifts its position toward the pyridinium group, adopting an S-type conformation, as shown in the P_220_ complex in Fig. 2B. This combination of intermolecular interactions, along with the reduction in the size of the macrocycle cavity, explains the decrease in energy toward the most stable structure. The energy decomposition analysis in Fig. 2D clearly shows a significant reduction in steric hindrance in P_220_ compared to earlier stages. At this point, the electrostatic contribution also becomes energetically favorable. Therefore, the formation of the lowest energy structure during the interlocking process results from the stabilization of both steric and electrostatic contributions.

**Fig. 3:**
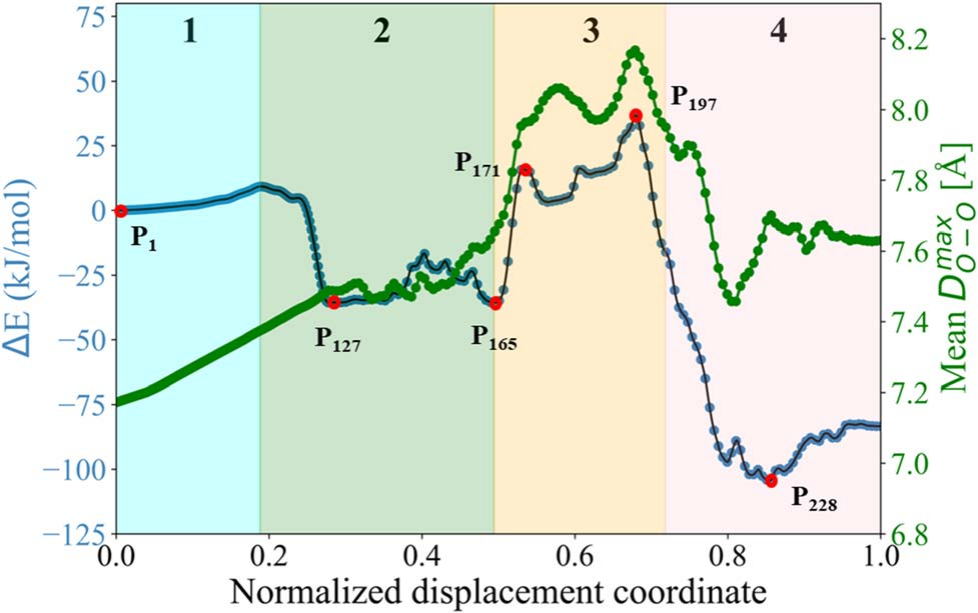
Energy profile (blue curve) and average maximum distance between oxygen atoms in 24C8 (*D*^max^_OO_, green curve) for the assembly of [1b⊂24C8]^2+^.

**Fig. 4:**
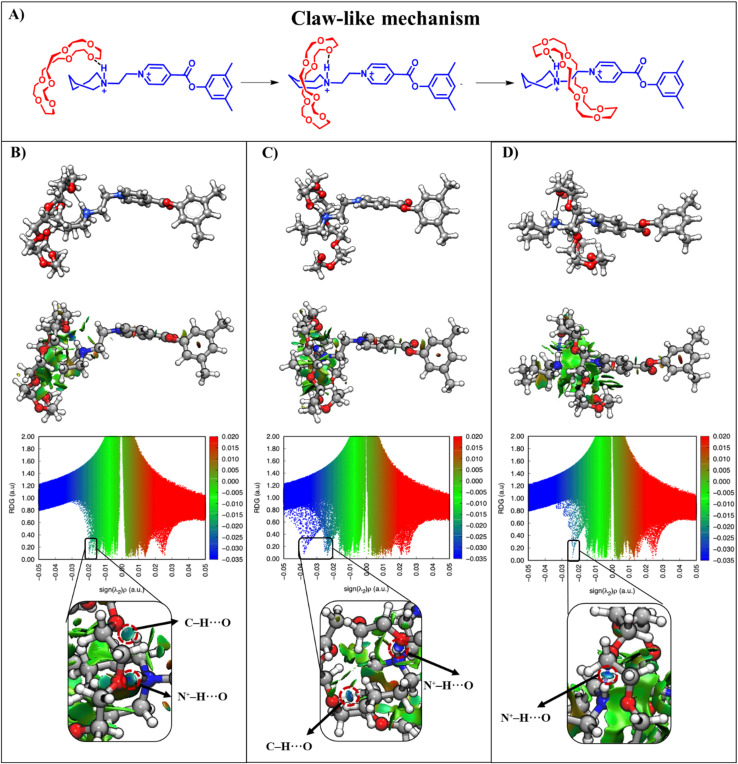
(A) Claw-like mechanism for the assembly process of complex [1b⊂24C8]^2+^, at structures (B) P_165_, (C) P_197_, and (D) P_228_ of the energy profile; NCI analysis of each structure of [1b⊂24C8]^2+^ and ball-and-stick models depicting the isosurfaces of 
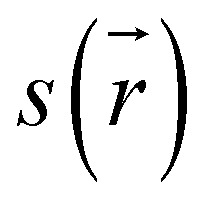
, with values of 
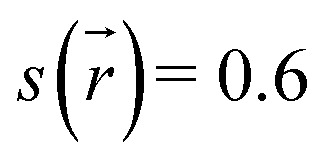
, colored according to the sign of 
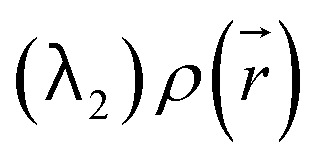
.

We now address the issue of steric hindrance in the assembly process by comparing two different doubly charged complexes: [1a⊂24C8]^2+^, with a piperidinium end-group, and [1b⊂24C8]^2+^, featuring an azepanium terminal group. As previously discussed, for complex [1a⊂24C8]^2+^, macrocycle expansion occurs at the preparation and activation stages, while the N^+^–H⋯O interaction appears until the activation stage, whereas complex [1b⊂24C8]^2+^ follows a more irregular path due to the bulkiness of the [1b]^2+^ terminal group. As shown in Fig. 3, the azepanium substituent markedly modifies the energy landscape, preventing the macrocycle from freely threading along the axle, unlike the situation with piperidinium (Fig. 2A and 3). As the macrocycle approaches the bulkier [1b]^2+^ terminus during the preparation stage, an important conformational change occurs, resulting in a claw-like arrangement (Fig. 4A), where the macrocycle surrounds the axle terminus, the azepanium end-group. As can be seen in Fig. 4B, this engulfed configuration aids threading by promoting the formation of N^+^–H⋯O and C–H⋯O hydrogen bonds, as demonstrated by the NCI analysis.

This mechanism permits the macrocycle to anchor to the axle and slide along [1b]^2+^. During the activation stage, it traverses the azepanium group, which adopts a boat conformation, as can be seen in structure P_197_ (Fig. 4C) stabilized by interactions with the 24C8 ring. In P_228_, only the N^+^–H⋯O hydrogen bond remains, as ring relaxation allows it to reposition around the ethyl group (Fig. 4D), resulting in the loss of the C–H⋯O interaction previously maintained by the azepanium group with the macrocycle. Throughout the stages of initialization, preparation, and stabilization, azepanium consistently adopts a twisted-boat conformation, as shown in P_228_. EDA-SBL analysis indicates that the steric contribution becomes negative and more significant compared to the reference; this is due to internal structural arrangements, where weak interactions and electrostatics relieve strain by relaxing both the macrocycle and the axle (Fig. S3C).

The claw-like mechanism, involving hydrogen-bond formation during the preparation stage, is not limited to systems with bulky terminal groups. A similar process also occurs in complexes with smaller terminal groups when a more rigid macrocycle is used. This is the case for the [1a⊂DB24C8]^2+^ complex. In this system, the presence of aromatic substituents on DB24C8 restricts the cavity size, introducing steric hindrance despite the smaller size of the piperidinium terminal group. The maximum average oxygen–oxygen distance *D*^max^_OO_ in DB24C8 is approximately 0.2 Å shorter than that in 24C8 in complexes with guest [1a]^2+^, based on the maximum values for each system. This reduction decreases the flexibility of the macrocycle, resulting in less smooth axle insertion. As a result, the ring is forced to approach the proton of the ammonium group, disrupting the original “sandwich-like” structure formed between the phenyl rings and the terminal group. This distortion allows hydrogen bonding, which stabilizes the macrocycle and helps it thread along the axle.

**Fig. 5:**
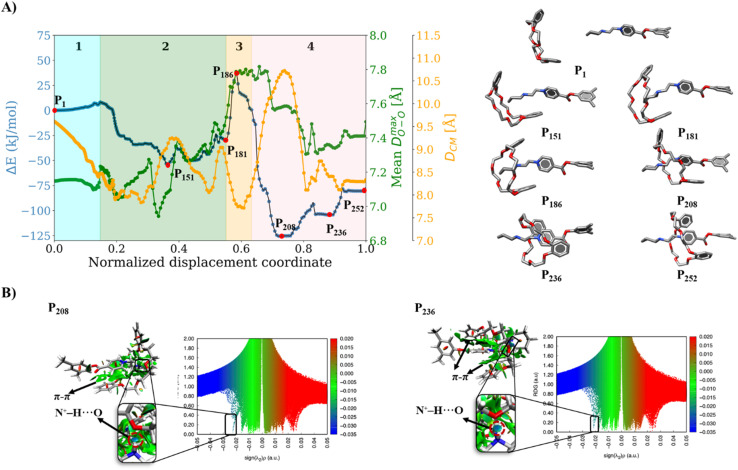
(A) Energy profile (blue curve), average maximum distance between oxygen atoms in DB24C8 (*D*^max^_OO_, green curve), and distances between the centers of mass of the aromatic rings (*D*_CM_, orange curve) for the assembly of [1a⊂DB24C8]^2+^ and structures P_1_, P_151_, P_181_, P_186_, P_208_, P_236_, and P_252_ corresponding to selected points along the curve. For clarity, hydrogen atoms are omitted. (B) Isosurfaces of the reduced density gradient with a value of colored according to sign(*λ*_2_) for the images P_208_ and P_236_.

The conformational rearrangement of the macrocycle as it passes through the terminal group is confirmed by measuring the distances between the centers of mass of the phenyl rings (*D*_CM_). After overcoming the energy barrier, the pyridinium unit on the guest undergoes a rotation that allows π-stacking interaction with one of the catechol rings of DB24C8, while the other remains anchored by a hydrogen bond to the piperidinium group. This dual anchoring defines the maximum separation between the aromatic rings, as shown in the orange curve of Fig. 5A, and stabilizes the lowest-energy structure through the combined contribution of hydrogen bonding and π–π stacking interactions. This is further supported by the NCI analysis, which reveals a sheet-like isosurface between the catechol and pyridinium groups in P_208_ (Fig. 5B). Additionally, the rotation of the piperidinium terminal group breaks the hydrogen bond present in the most stable conformation, allowing it to approach the pyridinium unit. This promotes the formation of new hydrogen bonds that favor the π–π stacking of both phenyl rings with the pyridinium group, as shown in P_236_ (Fig. 5B).

**Fig. 6:**
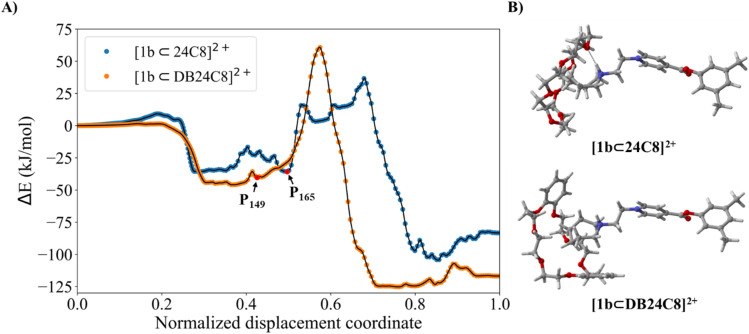
(A) Energy profiles for the self-assembly normalized displacement coordinate of [1b⊂24C8]^2+^ and [1b⊂DB24C8]^2+^. (B) representation of a structure corresponding to the [1b⊂24C8]^2+^ complex (P_165_), showing the hydrogen-bond interaction of the macrocycle, and the structure of the [1b⊂DB24C8]^2+^ complex at point P_149_, showing the opening of the macrocycle of DB24C8.

For the claw-like mechanism to occur, both the size of the terminal group and the flexibility of the macrocycle are crucial factors. When a bulky group like azepanium is combined with a rigid macrocycle like DB24C8, a smoother displacement coordinate is observed compared to the [1b⊂24C8]^2+^ complex (Fig. 6A). This difference arises because the rigidity of the macrocycle and the steric bulk of the terminal group prevent the formation of a hydrogen bond, resulting in a higher energy pathway. For complex [1b⊂DB24C8]^2+^, the macrocycle expands as the azepanium group approaches, adopting an L-shaped conformation (Fig. 6B), which restricts the maximum opening of DB24C8 (7.9 Å) compared to 24C8 (8.2 Å), highlighting the conformational constraint imposed by the catechol rings. As a result, the formation of a hydrogen bond between the azepanium proton and the phenolic oxygen atoms is hindered, favoring instead an interaction with one of the glycol chain oxygen atoms of DB24C8. This interaction enables guest rotation and promotes π–π stacking between the pyridinium group and one of the phenyl rings, contributing to the stabilization of the lowest-energy structure (Fig. S40).

**Fig. 7:**
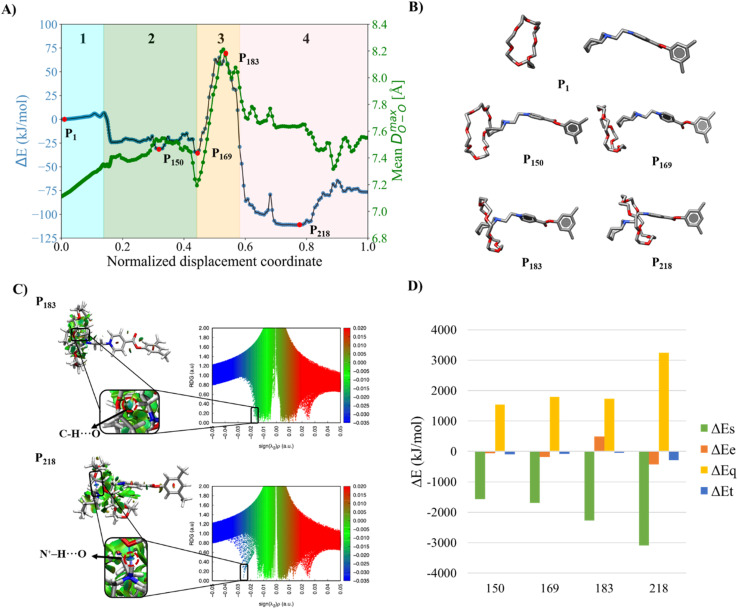
(A) Energy profile (blue curve), and average maximum distance between oxygen atoms in 24C8 (*D*^max^_OO_, green curve), for the assembly of [1c⊂24C8]^2+^. (B) Representative structures P_1_, P_150_, P_169_, P_183,_ and P_218_ of the assembly process of [1c⊂24C8]^2+^. For clarity, the hydrogen atoms are removed from the structures. (C) Isosurfaces of the reduced density gradient with a value of 
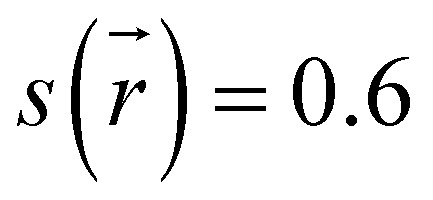
 colored according to sign(*λ*_2_) for the images P_183_ and P_218_. (D) EDA analysis for the corresponding images, showing the contributions to the energy, using P_1_ as reference.

The relationship between the rigidity of the macrocycle and the size of the terminal group becomes clearer when analyzing the assembly of the [1c⊂24C8]^2+^ complex. In this case, the assembly mechanism resembles that seen in systems with DB24C8 and azepanium; however, the increased flexibility of 24C8 allows for more efficient expansion to accommodate the steric hindrance caused by the terminal group. This is especially evident in the green curve of Fig. 7A, where the higher maximum value of 8.2 Å indicates the ability of the macrocycle to adapt more easily to the size and shape of the incoming azocanium group. The azocanium unit, which is even bulkier than azepanium, cannot form a hydrogen bond during the initial threading stage, preventing the claw-like mechanism and forcing the macrocycle to adopt the conformation shown in structure P_169_ (Fig. 7B). Along the displacement coordinate, the gradual adjustment of the O–C–C–O dihedral angles helps the macrocycle to expand, while the azocanium group maintains a twisted boat–chair geometry.

During the activation stage, an energy increase of 104 kJ mol^−1^ results from the expansion of the macrocycle in the absence of any significant stabilizing interactions. The P_183_ highest-energy structure is reached after a C–H⋯O hydrogen bond has formed between a methylene group on the azocanium ring and an oxygen atom from 24C8 (Fig. 7C). The interaction, together with the proximity of the 24C8 macrocycle to the ammonium group, halts the increase in energy and promotes the formation of a more stable N^+^–H⋯O hydrogen bond, resulting in a significant decrease in the total energy of the supramolecular complex.

Subsequently, the system adopts its most stable structure along the displacement coordinate, forming an S-type conformation. This geometry is stabilized through a combination of hydrogen bonds and electrostatic interactions occurring between the azocanium unit and the lower region of the 24C8 macrocycle. These interactions are evident from the NCI isosurfaces shown in Fig. 7C and the negative contribution to electrostatic energy seen in structure P_218_ (Fig. 7D). Notably, this S-type conformation consistently appears across all complexes using 24C8, indicating that, although the terminal group size affects the assembly pathway, it does not prevent the formation of the final stabilized geometry, thanks to the inherent flexibility of the macrocycle.

In contrast, efforts to model the assembly of the [1c⊂DB24C8]^2+^ complex using the same displacement-coordinate approach revealed significant limitations. The passage of the bulky azocanium group through the rigid DB24C8 macrocycle caused multiple bond reorganizations within the ring, even breaking some bonds in the macrocycle, and producing erratic energy profiles. These results indicate that the combined steric hindrance from the terminal group and the limited expansion capacity of DB24C8, where *D*^max^_OO_ does not exceed 7.9 Å, hinders the effective accommodation of the guest. Consistently, there is no experimental evidence for the formation of the [1c⊂DB24C8]^2+^ complex, which further supports the interpretation that its assembly is not feasible without rupture of the macrocycle. Overall, these findings emphasize that macrocycle flexibility and the size of the terminal group are critical factors governing supramolecular assembly. Moreover, they underscore the essential roles of steric effects and electrostatic contributions in enabling key hydrogen-bonding interactions that influence the viability and energetics of the interlocking process.

To examine the role of electrostatic interactions, energy curves were calculated for two additional axles, specifically, [2]^+^ and [3]. Guest [2]^+^ was built by replacing the piperidinium group with a cyclohexyl group, while guest [3] involved substituting both positively charged nitrogen atoms with neutral carbon atoms. Note that all axles and complexes are isoelectronic.

**Fig. 8:**
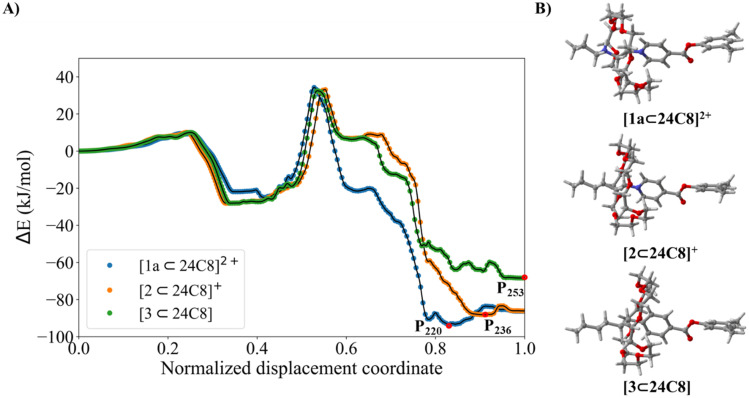
(A) Energy profiles of the self-assembly process obtained with NEB/ANI-1ccx for [1a⊂24C8]^2+^, [2⊂24C8]^+^, and [3⊂24C8]. (B) P_220_, P_236_ and P_253_ global minima structures for each system, respectively.

The NEB/ANI-1ccx profiles are shown in Fig. 8A. As can be seen, the activation energy barriers for the three systems do not exhibit significant changes until they reach the transition state. However, upon overcoming the transition state, the energy profiles for the complexes [2⊂24C8]^+^ and [3⊂24C8] differ significantly from that of complex [1a⊂24C8]^2+^. Although a valley is also formed for systems with cyclohexyl, for NDC values around 0.6, structures of higher energy than those obtained for the piperidinium complex were found. This is due to their inability to form stabilizing hydrogen bonds between the macrocycles and the axle. The energies of the structures in these valleys are approximately 42 kJ mol^−1^ higher than that corresponding to the [1a⊂24C8]^2+^ complex.

Recognition of the charge variations among the three rotaxanes is consistent with the changes in stability of the global minima. These minima correspond to the structures illustrated in Fig. 8B, which are located at points P_220_, P_236_, and P_253_ along the normalized displacement coordinate for each axle. The first feature to be noted is that the predicted most stable structure is the doubly charged axle, followed by the singly charged axle, and finally, the minimum with the highest energy is that with a neutral axle. Rotaxane [2⊂24C8]^+^ is 19 kJ mol^−1^ higher in energy than the doubly charged complex [1a⊂24C8]^2+^, while neutral [3⊂24C8] is 30 kJ mol^−1^ higher. The observed stability trend [3⊂24C8] < [2⊂24C8]^+^ < [1a⊂24C8]^2+^ strongly suggests that the electrostatic interactions between axles and macrocycles are fundamental for the stabilization of these rotaxanes.

The energy profiles for different axles demonstrate the ability of the ANI-1ccx potential to distinguish the charge variations in the three rotaxanes. This finding is significant, considering that ANI-1ccx does not include charged structures in its training set. We attribute this to the architecture and the training of the ANI-1ccx model. Regarding the architecture, one can mention that the input of the ANI models uses symmetry functions, based on the Behler–Parrinello scheme,^61^ to describe the local atomic environment in a translationally and rotationally invariant way, and that each element has its own neural network, mapping its atomic environment to an energy contribution. Regarding training, by keeping the DFT-trained early layers and training the final layers with the CCSD(T)*/CBS global standard accuracy, overfitting is avoided due to the small size of the gold-standard training set.

The energy decomposition analyses EDA-SBL of structures P_220_, P_236_, and P_253_, corresponding to the energy minima of the [1a⊂24C8]^2+^, [2⊂24C8]^+^, and [3⊂24C8] complexes, are shown in Fig. 2D, S1C, and S2C, respectively. These plots show that at the global minima, the electrostatic energy contributions in the charged complexes are negative, with the doubly charged system having an electrostatic contribution of almost twice that of the singly charged rotaxane, and the neutral system having an almost vanishing (positive) electrostatic contribution. This result is perfectly aligned with the energy differences obtained from the ANI-1ccx energy profiles. Thus, we can conclude that the methodology NEB/ANI-1ccx correctly predicts the stability of rotaxanes as a function of their electrical charge, even though the training set of this NNP did not contain charged systems.

Finally, to complement these analyses with an energetic description of the association process, we evaluated the activation energy barriers for all systems. Table 1 shows the estimated barriers and the calculation times for convergence, demonstrating good agreement in the ordering and similarity with the experimental free energies, even though the barriers were calculated without considering the effects of temperature or solvent, effects that are certainly important and that could modify the trajectory and the energy profile and therefore the agreement shown in Table 1. These values were obtained directly from the NEB/ANI-1ccx profiles as the energy difference between the maximum in the activation stage and the minimum in the preparation stage.

**Table 1:** Experimental free energy values and potential energies obtained from NEB/ANI-1ccx calculations, along with the partial (NEB1 and NEB2) and total convergence times, in hours, of the NEB segments composing energy profiles. Note that there are no timings for macrocycle breaking because convergence is not achieved at certain points along the profile^a^

Complex	Δ*G*^‡^_exp_ (kJ mol^−1^)^b^	Δ*E*^‡^ (kJ mol^−1^)	*t*_NEB1_ (h)	*t*_NEB2_ (h)	*t*_total_ (h)
[1a⊂24C8]^2+^	<60.0	59.2	0.04	3.90	3.94
[1b⊂24C8]^2+^	71.7	72.3	0.02	16.8	16.8
[1c⊂24C8]^2+^	>100	105.0	0.06	19.7	19.7
[1a⊂DB24C8]^2+^	74.2	89.6	0.41	39.5	39.9
[1b⊂DB24C8]^2+^	92.8	107.0	0.02	3.80	3.82
[1c⊂DB24C8]^2+^	NF	MB	—	—	—
[2⊂24C8]^+^	NR	61.2	0.03	5.70	5.73
[3⊂24C8]	NR	60.4	0.03	5.50	5.53

a All calculations were performed using an NVIDIA A100 GPU (80 GB PCIe); NF: No formation; MB: macrocycle breaking; NR: no report in the literature.

b References Δ*G*^‡^_exp_ values.^43,44^

The values in the table are accompanied by the total computational times required by each NEB segment forming the respective energy profile to reach the convergence criterion. The first NEB segment (P_1_ to P_127_), labeled NEB1, converges quickly, as it describes only the approach of the macrocycle to the guest, while the second NEB (P_127_ to P_253_), labeled NEB2, is the most computationally intensive because it involves the assembly process, reflecting the complexity of the potential energy surface observed in previous profiles. This second step requires more images to obtain detailed mechanistic insight and improve the approximation to the minimum energy path.

Currently, the level of detail discussed in the previous paragraphs can only be achieved using the NEB/ANI-1ccx methodology presented in this work, because the large number of images needed makes it unfeasible with DFT, and even more challenging with the much more demanding CCSD(T). Therefore, the results presented here provide a detailed description of the assembly process and the interactions and energy contributions involved in forming the rotaxane-like complexes, at a very reasonable computational cost, as can be seen in Table 1. Therefore, this work demonstrates the potential of integrating well-trained and meticulously designed machine learning potentials with established yet computationally intensive algorithms to investigate the potential energy surface of complex systems, such as supramolecular assembly, and to elucidate the underlying mechanisms governing system dynamics.

## Conclusions

This work shows that by combining the nudged elastic band (NEB) method with the deep neural network ANI-1ccx, it is possible to obtain energy profiles where the energy of a supramolecular complex is plotted against the normalized displacement coordinate (NDC) produced by the NEB algorithm. These profiles are very useful for understanding the interlocking mechanisms that drive the assembly of rotaxanes. The quality of the energies and forces supplied by the ANI-1ccx potential, together with the low computational times required to obtain them, opens up the possibility of generating energy profiles with a resolution that is unachievable using traditional methods like force fields or all-electron calculations, which are at least six orders of magnitude slower and more computationally demanding. In this study, we have presented energy profiles with 250 images, obtained in two segments, which are impossible to attain with DFT and even less feasible with highly correlated wave function methods.

The protocol proposed in this work reveals four energy profile stages: initialization, preparation, activation, and stabilization. Notably, ANI-1ccx effectively describes structural changes in interlocking mechanisms forming supramolecular complexes, especially rotaxanes. This transferable machine-learning model for predicting molecular energies and forces is effective, thanks to three features: it was trained on high-quality CCSD(T)*/CBS data from a smaller set selected from a larger DFT dataset; it encodes local atomic environments invariantly to rotation, translation, and permutation, essential for transferability and physical accuracy—especially for charged atoms, despite not including charged species in training; and it features a deep network structure that captures complex non-linear relationships in the potential energy surface, like those in supramolecular assembly. These features enable rapid calculations many times faster than CCSD(T)*, with high accuracy, as shown by NEB calculations.

The information gathered from the NDC helped us understand how the size and charge of the terminal group on the guests and the rigidity of the macrocycle influence the energy barrier during assembly. It also helped identify the main interactions that promote macrocycle insertion. By comparing Shubin Liu's energy decomposition analysis of piperidinium and cyclohexyl complexes using 24C8 as the macrocycle, we have shown that electrostatic and steric contributions are essential for reaching the global minima. Additionally, this method allowed us to establish the existence of two key steps during the assembly process under the electrostatically assisted sliding approach, even though, as mentioned above, the ANI-1ccx potential does not include charged systems in its training set. This approach also enabled us to determine the potential energy barriers for threading processes, with an average difference of 8 kJ mol^−1^ compared to the free energy barriers reported in the literature.

In summary, this work paves the way for future research in using machine learning to address complex situations such as supramolecular assembly, where no chemical bonds are broken or formed, and elucidate different mechanisms that govern system dynamics. Important effects to consider in future work include solvent and temperature when predicting free energies.

## Author contributions

All authors contributed equally to the conceptualization of the project and the manuscript's design, writing, editing, and revision.

## Conflicts of interest

There are no conflicts to declare.

## Supplementary Material

SC-OLF-D5SC08303F-s001

SC-OLF-D5SC08303F-s002

## Data Availability

The data supporting this article have been included as part of the supplementary information (SI). Supplementary information: A description of (1) the non-interacting systems limit, (2) the energy profiles and EDA-SBL for some of the supramolecular complexes discussed in text, (3) the non-covalent interactions for some of the supramolecular complexes discussed in text, (4) plots of the energy and the distances between the center of mass of the phenyl rings in the [1a⊂DB24C8]^2+^ complex, and (5) the S-type conformations of rotaxanes, are available in the file Supporting_Information-LPZ-AV-JT.pdf. The animations of the assembly of [1a⊂24C8]^2+^ and [1a⊂DB24C8]^2+^, together with the Cartesian coordinates of 8 representative structures discussed in the text, are available in the compressed file Animation-Coordinates-LPZ-AV-JT.zip. See DOI: https://doi.org/10.1039/d5sc08303f.
