# Supplementary material for: The interlocking process in molecular machines explained by a combined approach: the nudged elastic band method and machine learning potential
Source: Chem Sci. 2026 Jan 13;17(12):6055–67. doi: 10.1039/d5sc08303f (PMC12857636; doi:10.1039/d5sc08303f)
Supplement: SC-017-D5SC08303F-s002 [file SC-017-D5SC08303F-s002.pdf]

Supplementary Information (SI) for Chemical Science.
This journal is © The Royal Society of Chemistry 2026

# Supporting Information

# The interlocking process in molecular machines explained by a combined approach: the nudged elastic band method and a machine learning potential

*Lucio Peña-Zarate, Alberto Vela* and Jorge Tiburcio**

Department of Chemistry, Centro de Investigación y de Estudios Avanzados (Cinvestav), Avenida IPN 2508, 07360 Mexico City, Mexico.

*Corresponding authors: avela@cinvestav.mx and jtiburcio@cinvestav.mx

**Contents**

**1. Non-interacting systems limit** .......... 2
**2. Energy profiles and EDA-SBL** .......... 3
**2.1.** Shubin Liu's energy decomposition analysis (EDA-SBL) .......... 3
**2.2.** Energy profiles and the maximum mean distance between the O-O of the macrocycle for each supramolecular system .......... 3
**3. Non-covalent interactions (NCI)** .......... 9
**3.1.** Parameters of NCI .......... 10
**4. The curve of energy and centers of mass distance (CMD)** .......... 22
**5. Conformation of rotaxane** .......... 23
**6. References** .......... 24

## 1. Non-interacting systems limit

The non-interacting limit was estimated by subtracting the sum of the single-point energies of the isolated components (each in the same geometry as in the complex) from the single-point energy of the combined system, where both components are placed together in a single .xyz file without any chemical bonding. All energies were obtained using the ANI-1ccx potential. This approach provides an estimate of the interaction energy arising purely from bringing the two subsystems into proximity, without accounting for structural relaxation or bonding effects. This procedure guarantees that $\mathbf{P_1}$ indeed corresponds to a non-interacting complex.

**Table S1**. Values of the energy differences obtained from the $\mathbf{P_1}$ structure concerning the sum energy of its components (**axle** and **macrocycle**) for each system, respectively.

| **Axle** | **$[1]^{2+}$+ 24C8 (kJ/mol)** | **$P_1$ (kJ/mol)** | **ΔE (kJ/mol)** |
|---|---|---|---|
| **a** | $-6.05\times10^6$ | $-6.05\times10^6$ | 0 |
| **b** | $-6.16\times10^6$ | $-6.16\times10^6$ | 0 |
| **c** | $-6.26\times10^6$ | $-6.26\times10^6$ | 0 |
| | **$[1]^{2+}$+ DB24C8** | **$P_1$** | **ΔE** |
| **a** | $-6.85\times10^6$ | $-6.85\times10^6$ | 0 |
| **b** | $-6.96\times10^6$ | $-6.96\times10^6$ | 0 |
| | **$[2]^{1+}$+ 24C8** | **$P_1$** | **ΔE** |
| **$[2]^{+}$** | $-6.01\times10^6$ | $-6.01\times10^6$ | 0 |
| | **[3] + 24C8** | **$P_1$** | **ΔE** |
| **[3]** | $-5.97\times10^6$ | $-5.97\times10^6$ | 0 |

## 2. Energy profiles and EDA-SBL

### 2.1. Shubin Liu's energy decomposition analysis (EDA-SBL) **Δ**

This analysis reveals how steric, electrostatic, and quantum effects influence supramolecular assembly.[1] This approach assumes that the total energy of an atom or molecule can be partitioned as.

$$E[\rho] = E_s[\rho] + E_e[\rho] + E_q[\rho] \quad , \tag{1}$$

where $E_s[\rho]$ is the steric term, and assumed to come from the Weizsäcker contribution to the kinetic energy functional, namely,

$$E_s[\rho] = T_W[\rho] = \frac{1}{8}\int \frac{|\nabla\rho(r)|^2}{\rho(r)} dr \quad . \tag{2}$$

The term $E_e[\rho]$ is the electrostatic contribution, given by,

$$E_e[\rho] = V_{ne}[\rho] + J[\rho] + V_{nn}[\rho] \quad , \qquad (3$$

where $V_{ne}[\rho]$ is the electron-nuclear attraction, $J[\rho]$, the direct interelectronic Coulomb interaction, and $V_{nn}[\rho]$, the nuclear-nuclear repulsion.

Finally, the last term $E_q[\rho]$ is the quantum energy, which is given by,

$$E_q[\rho] = E_{Pauli}[\rho] + E_{XC}[\rho] \quad . \tag{4}$$

The first term is the Pauli energy, defined as $E_{Pauli}[\rho] = T_S[\rho] - T_W[\rho]$, where $T_S[\rho]$ is the kinetic energy of non-interacting electrons and $T_W[\rho]$ is the Weizsäcker kinetic energy, and $E_{XC}[\rho]$, is the exchange-correlation energy.[2,3]

### 2.2. Energy profiles and the maximum mean distance between the O-O of the macrocycle for each supramolecular system

Figure S1-S4 represents the energy profiles (blue) built from two NEB/ANI-1ccx calculations containing 125 intermediate images each. These are divided into 4 stages that describe the assembly process, The first stage (Initialization) represents the free species, the second stage (Preparation) is the interaction between the axis and the free macrocycle, the

third stage (Activation) is the sliding process through the terminal group of the axis and finally, the fourth stage (Stabilization) is the formation of the most stable structure.

The green curve represents the average of the maximum distances between oxygen pairs in the macrocycle, which allows for determining the changes in the expansion of the macrocycle along the normalized displacement coordinate.

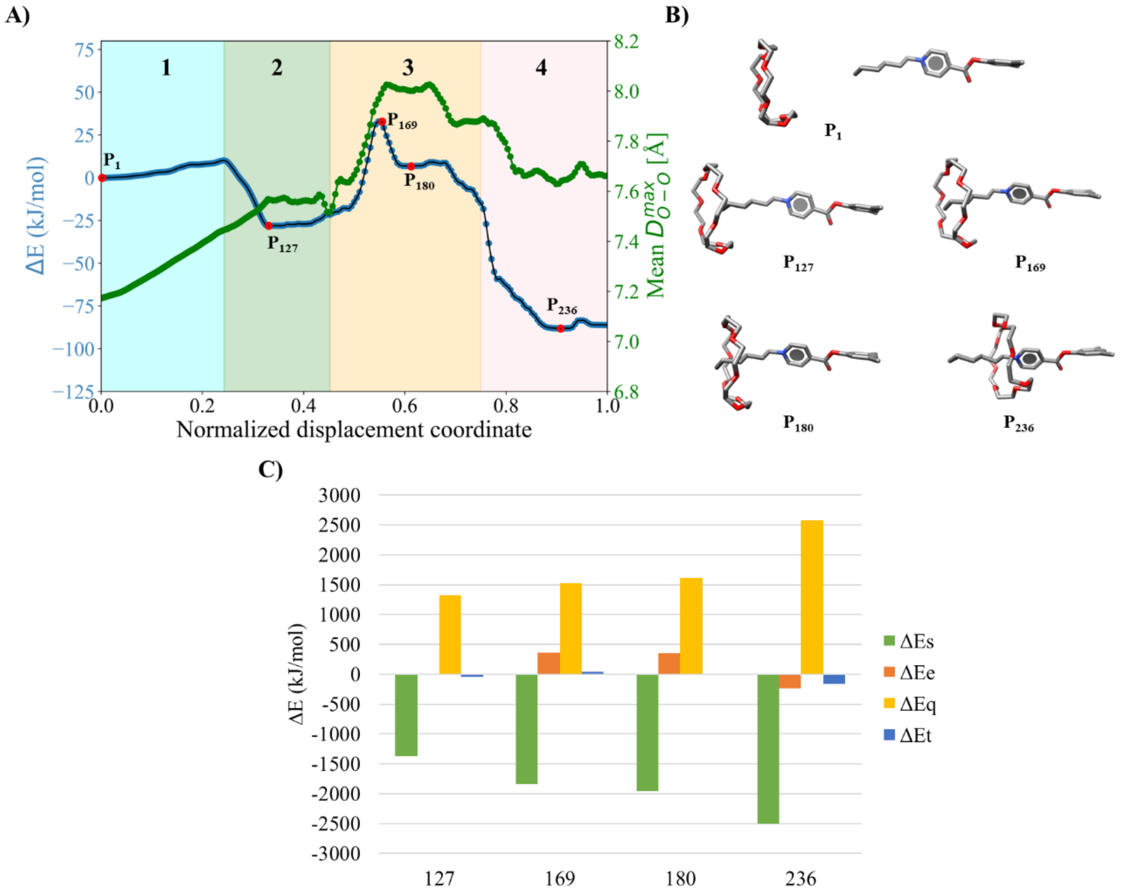


**Figure S1**. Energy profile (or curve) and mean maximum distance between the **24C8** oxygens for the normalized displacement of the assembly of **A) [2⊂24C8]$^+$**, **B)** representative structures of each stage in the assembly process in which hydrogens were removed for simplicity and **C)** bar graph of Shubin Liu's energy decomposition analysis of the energy difference taking **$P_1$** as reference for **$P_{127}$**, **$P_{169}$**, **$P_{180}$**, and **$P_{236}$**.

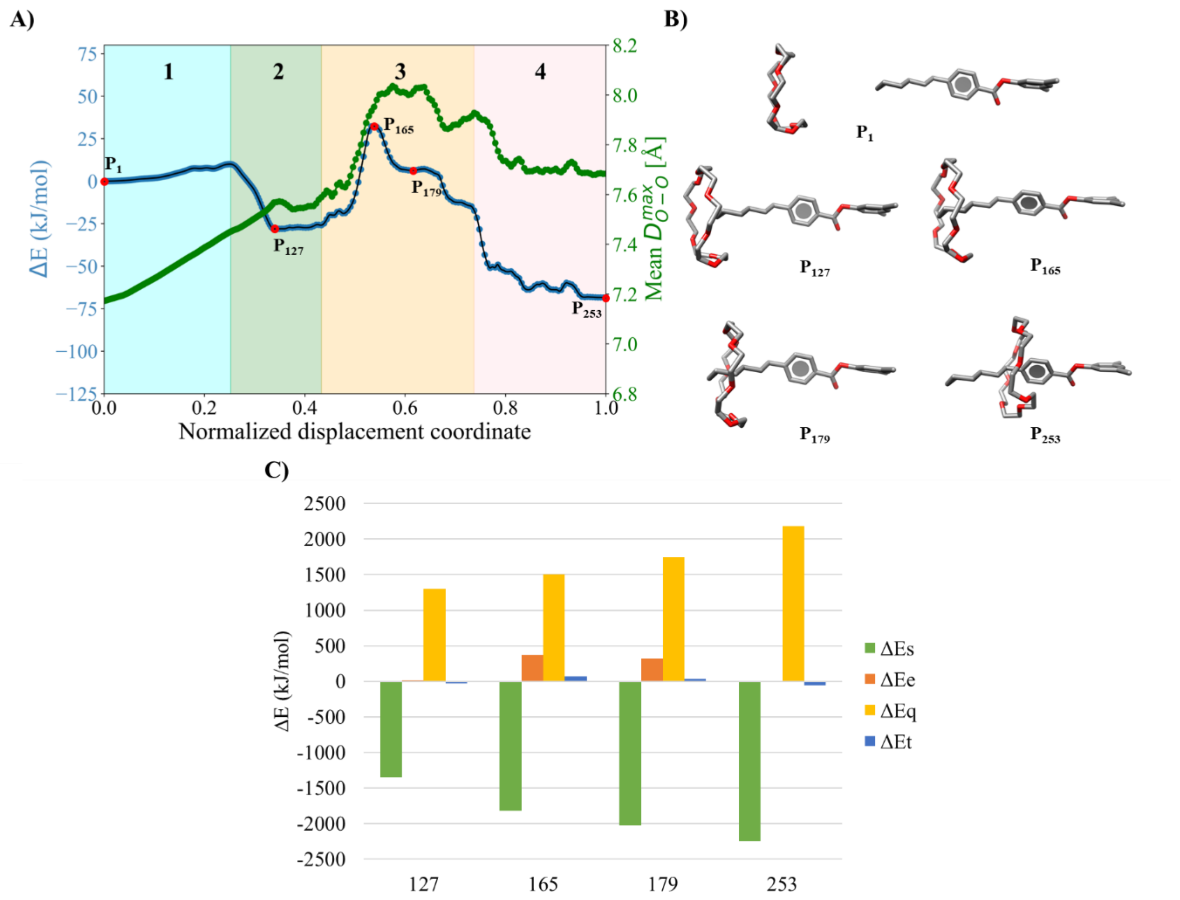


**Figure S2**. Energy profile (or curve) and mean maximum distance between the **24C8** oxygens for the normalized displacement of the assembly of **A) [3⊂24C8]**, **B)** representative structures of each stage in the assembly process in which hydrogens were removed for simplicity and **C)** bar graph of Shubin Liu's energy decomposition analysis of the energy difference taking $\mathbf{P_1}$ as reference for $\mathbf{P_{127}}$, $\mathbf{P_{165}}$, $\mathbf{P_{179}}$, and $\mathbf{P_{253}}$.

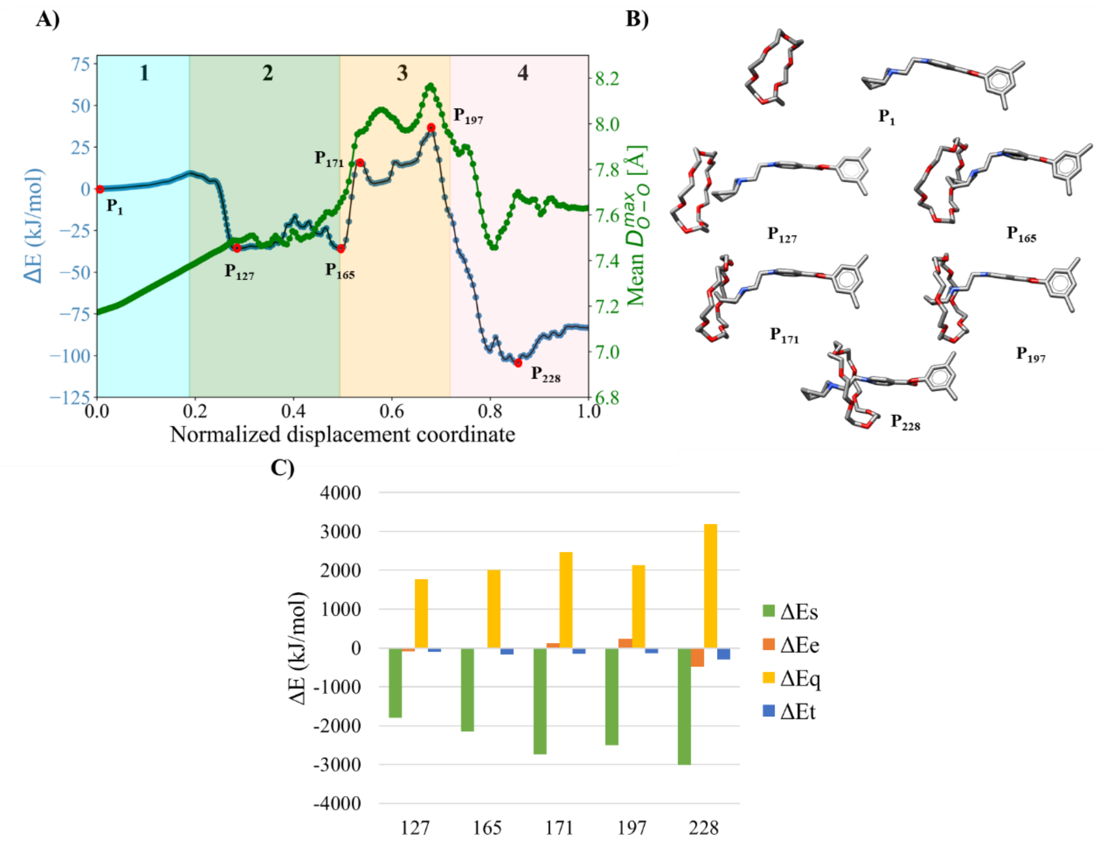


**Figure S3**. Energy profile (or curve) and mean maximum distance between the **24C8** oxygens for the normalized displacement of the assembly of **A) [1b⊂24C8]**$^{2+}$, **B)** representative structures of each stage in the assembly process in which hydrogens were removed for simplicity and **C)** bar graph of Shubin Liu's energy decomposition analysis of the energy difference taking **P**$_1$ as reference for **P**$_{127}$, **P**$_{165}$, **P**$_{171}$, **P**$_{197}$, and **P**$_{228}$.

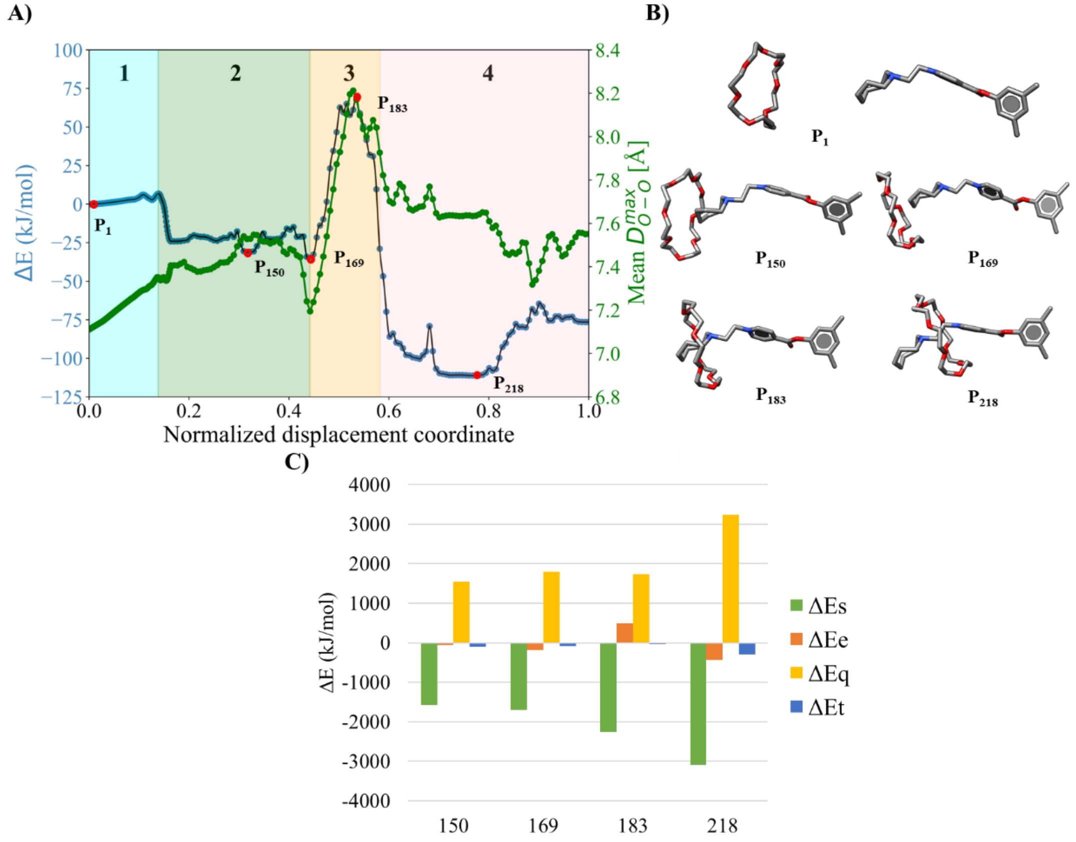


**Figure S4**. Energy profile (or curve) and mean maximum distance between the **24C8** oxygens for the normalized displacement of the assembly of **A)** $\mathbf{[1c\subset 24C8]^{2+}}$ **B)** representative structures of each stage in the assembly process in which hydrogens were removed for simplicity and **C)** bar graph of Shubin Liu's energy decomposition analysis of the energy difference taking $\mathbf{P_1}$ as reference for $\mathbf{P_{150}}$, $\mathbf{P_{169}}$, $\mathbf{P_{183}}$ and $\mathbf{P_{218}}$.

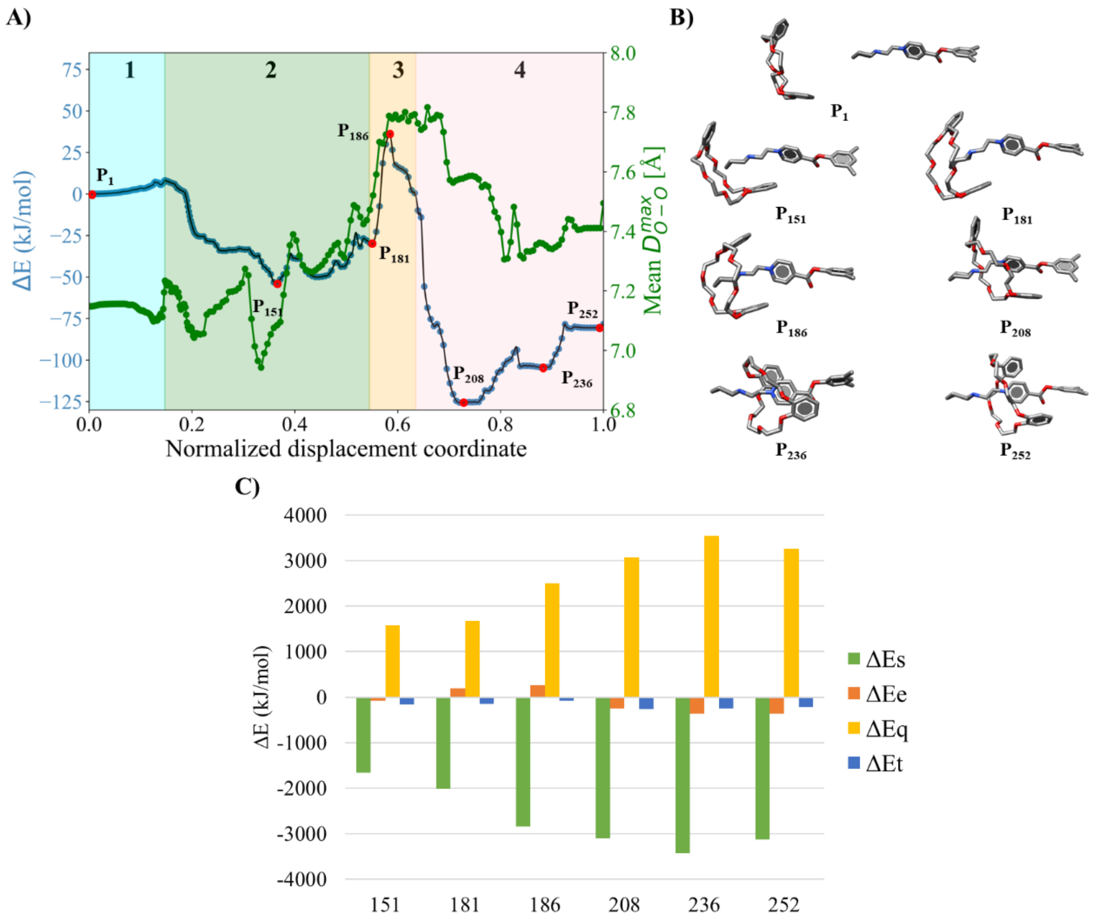


**Figure S5**. Energy profile (or curve) and mean maximum distance between the **DB24C8** oxygens for the normalized displacement of the assembly of **A)** **[1a⊂DB24C8]**$^{2+}$, **B)** representative structures of each stage in the assembly process in which hydrogens were removed for simplicity and **C)** bar graph of Shubin Liu's energy decomposition analysis of the energy difference taking $\mathbf{P_{1}}$ as reference for $\mathbf{P_{151}}$, $\mathbf{P_{181}}$, $\mathbf{P_{186}}$, $\mathbf{P_{208}}$, $\mathbf{P_{236}}$, and $\mathbf{P_{252}}$.

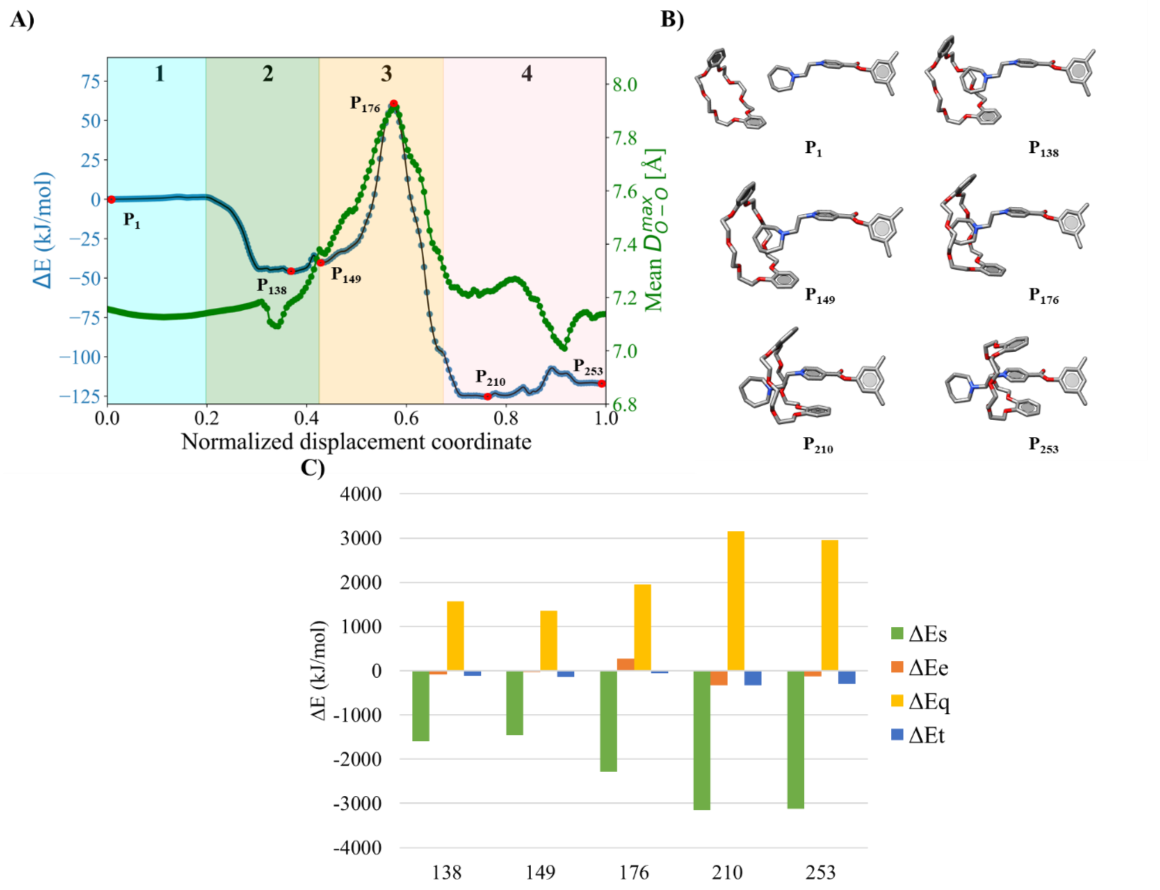


**Figure S6**. Energy profile (or curve) and mean maximum distance between the **DB24C8** oxygens for the normalized displacement of the assembly of **A) [1b⊂DB24C8]$^{2+}$**, **B)** representative structures of each stage in the assembly process in which hydrogens were removed for simplicity and **C)** bar graph of Shubin Liu's energy decomposition analysis of the energy difference taking $\mathbf{P_1}$ as reference for $\mathbf{P_{138}}$, $\mathbf{P_{149}}$, $\mathbf{P_{176}}$, $\mathbf{P_{210}}$, and $\mathbf{P_{253}}$.

### 3. Non-covalent interactions (NCI)

To study the assembly interactions, non-covalent interactions (NCI) analyses were done. NCI uses the reduced density gradient, the dimensionless quantity $s = 1/(2(3\pi^2)^{1/3}|\nabla\rho|/\rho^{4/3}$, which is highly important in DFT as it is a local measure of the deviations of the electron density $\rho$ from the homogeneous electron behavior.[4]

NCI helps distinguish covalent and non-covalent interactions by combining the values of the reduced density gradient in the neighborhood of a bond critical point, or where the reduced density gradient is small, and the second eigenvalue, $\lambda_2$, of the laplacian of the density at the corresponding point. The sign of $\lambda_2$ distinguishes between bonded ($\lambda_2 < 0$) and non-bonded ($\lambda_2 > 0$) interactions, helping to distinguish between types of non-covalent interactions, while the density itself provides information about their strength.[5,6]

**3.1.** Parameters of NCI

Figure S7-S42 reports the results of the NCI obtained for the representative structures of each stage of the energy profiles, from which the geometries for the single-point calculation with DFT were obtained with the theory model. PBE0-D3/def2TZVP in the Gaussian 09 program, to later perform the NCI calculation with a grid x,y,z of 0.10 0.10 0.10, in the program. Multiwfn, in Diagram 1, displays the color representation for interpreting non-covalent interactions in the NCI.[7]

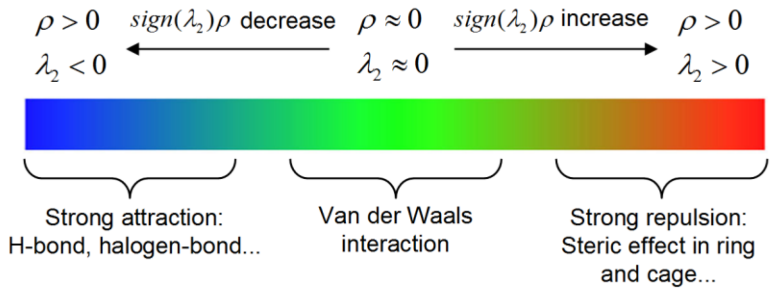


**Diagram 1**. Representation of the product of sign of ($\lambda_2$) and $\rho(\vec{r})$, through colors for the identification of non-covalent interactions.

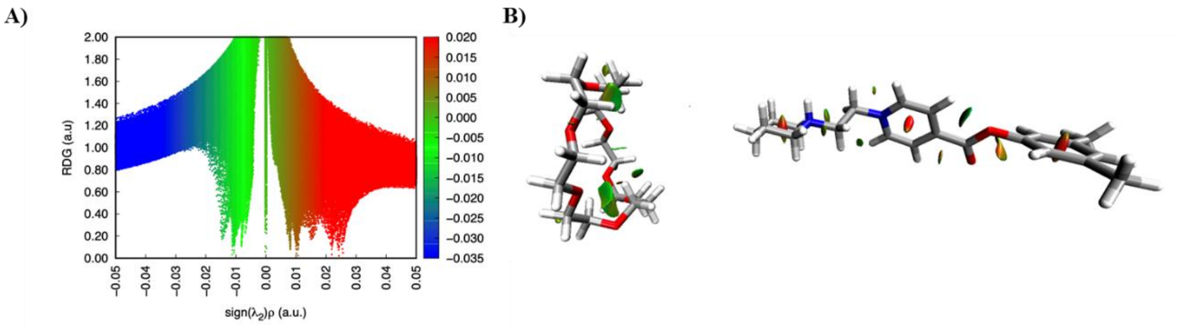

**Figure S7**. Standard NCI index representations of position $\mathbf{P_1}$ taken from the 253-point energy profile of the **[1a⊂24C8]$^{2+}$** complex in **A)** $s(\vec{r})$ plotted against the sign $(\lambda_2)\rho(\vec{r})$. **B)** Isosurfaces of $s(\vec{r})$ = 0.6 colored by sign $(\lambda_2)\rho(\vec{r})$.

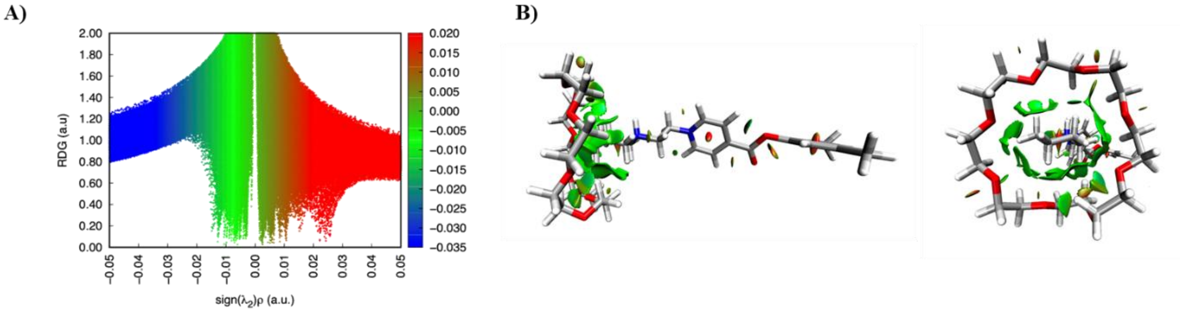


**Figure S8**. Standard NCI index representations of position $\mathbf{P_{127}}$ taken from the 253-point energy profile of the **[1a⊂24C8]$^{2+}$** complex in **A)** $s(\vec{r})$ plotted against the sign $(\lambda_2)\rho(\vec{r})$. **B)** Isosurfaces of $s(\vec{r})$ = 0.6 colored by sign $(\lambda_2)\rho(\vec{r})$.

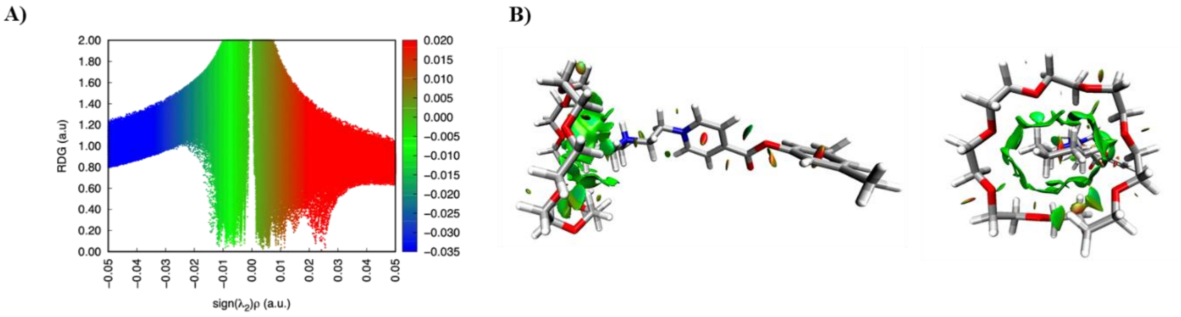


**Figure S9**. Standard NCI index representations of position $\mathbf{P_{140}}$ taken from the 253-point energy profile of the **[1a⊂24C8]$^{2+}$** complex in **A)** $s(\vec{r})$ plotted against the sign $(\lambda_2)\rho(\vec{r})$. **B)** Isosurfaces of $s(\vec{r})$ = 0.6 colored by sign $(\lambda_2)\rho(\vec{r})$.

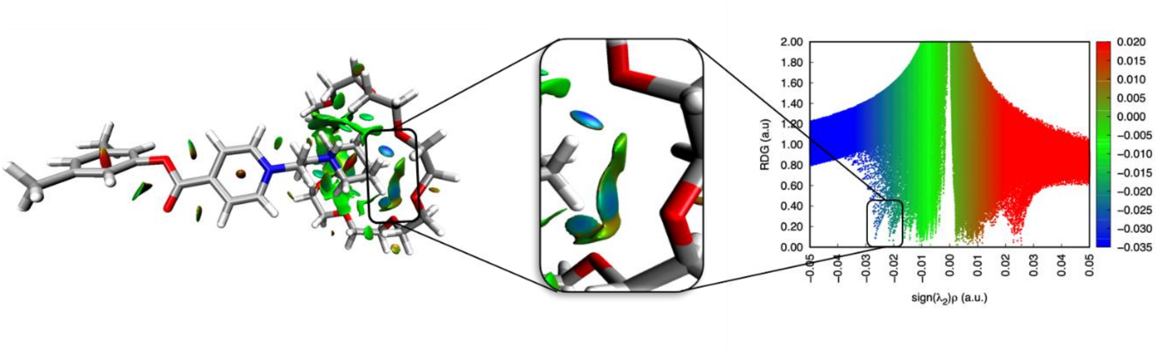

**Figure S10**. Standard NCI index representations of position $\mathbf{P_{162}}$ taken from the 253-point energy profile of the $\mathbf{[1a{\subset}24C8]^{2+}}$ complex of $s(\vec{r})$ plotted against the sign $(\lambda_2)\rho(\vec{r})$ and isosurfaces of $s(\vec{r})$ = 0.6 colored by sign $(\lambda_2)\rho(\vec{r})$.

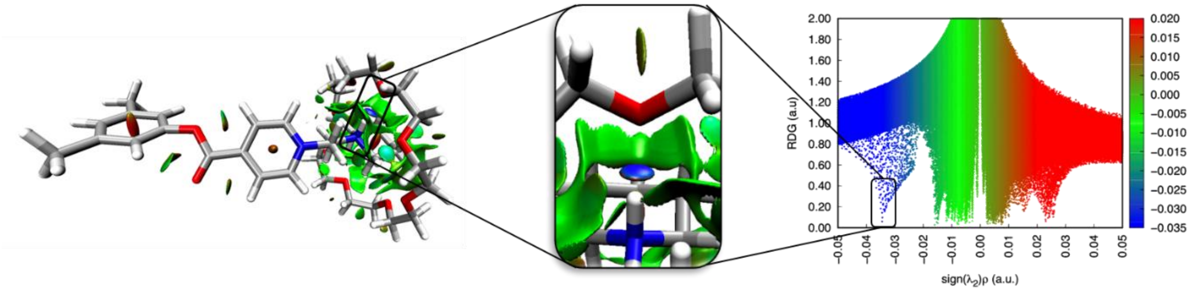


**Figure S11**. Standard NCI index representations of position $\mathbf{P_{181}}$ taken from the 253-point energy profile of the $\mathbf{[1a{\subset}24C8]^{2+}}$ complex of $s(\vec{r})$ plotted against the sign $(\lambda_2)\rho(\vec{r})$ and isosurfaces of $s(\vec{r})$ = 0.6 colored by sign $(\lambda_2)\rho(\vec{r})$.

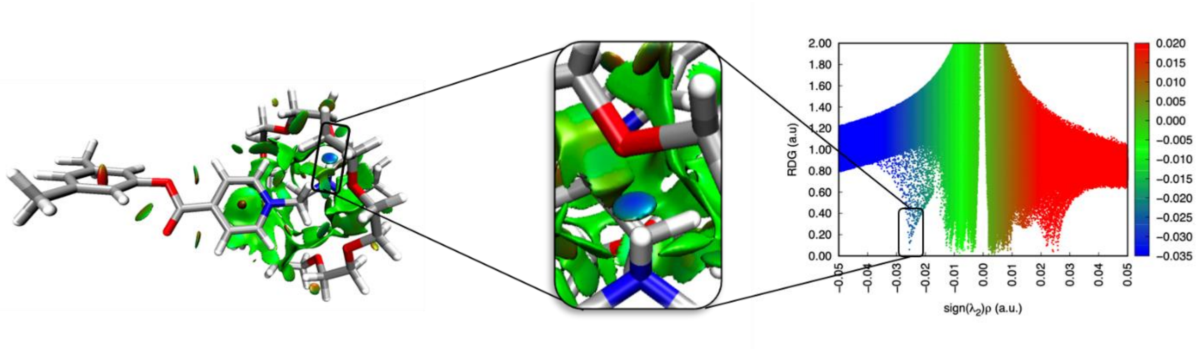


**Figure S12**. Standard NCI index representations of position $\mathbf{P_{220}}$ taken from the 253-point energy profile of the $\mathbf{[1a{\subset}24C8]^{2+}}$ complex of $s(\vec{r})$ plotted against the sign $(\lambda_2)\rho(\vec{r})$ and isosurfaces of $s(\vec{r})$ = 0.6 colored by sign $(\lambda_2)\rho(\vec{r})$.

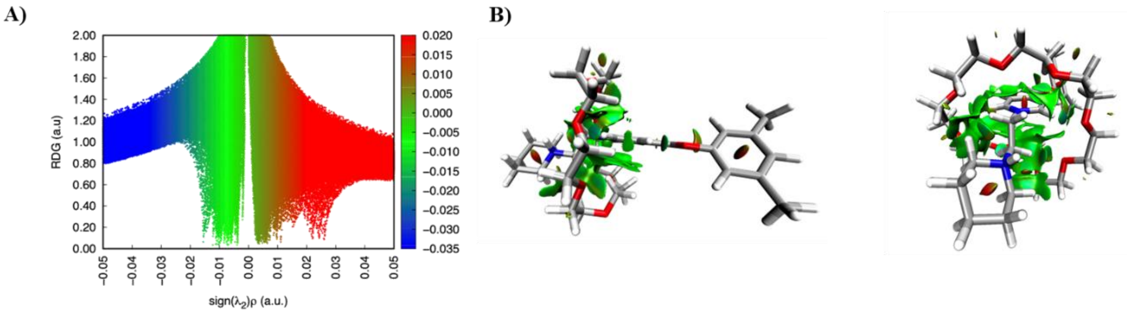

**Figure S13**. Standard NCI index representations of position $\mathbf{P_{253}}$ taken from the 253-point energy profile of the **[1a⊂24C8]**$^{2+}$ complex in **A)** $s(\vec{r})$ plotted against the sign $(\lambda_2)\rho(\vec{r})$. **B)** Isosurfaces of $s(\vec{r})$ = 0.6 colored by sign $(\lambda_2)\rho(\vec{r})$.

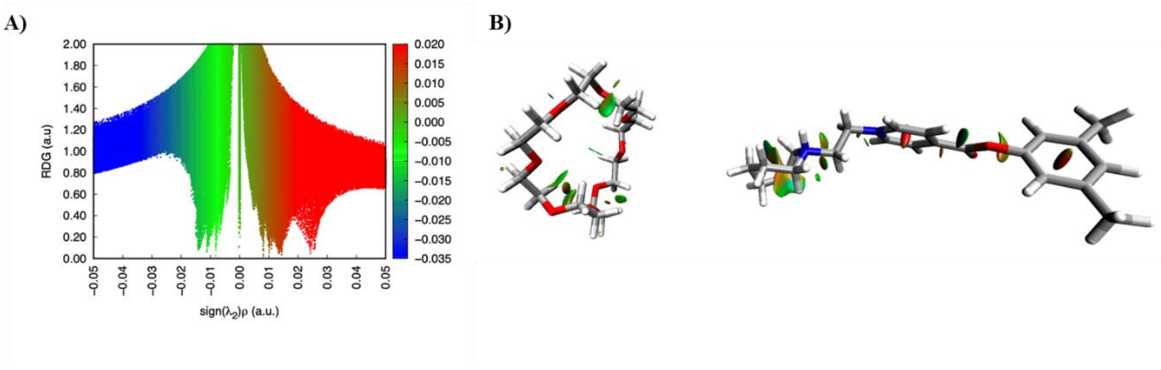


**Figure S14**. Standard NCI index representations of position $\mathbf{P_1}$ taken from the 253-point energy profile of the **[1b⊂24C8]**$^{2+}$ complex in **A)** $s(\vec{r})$ plotted against the sign $(\lambda_2)\rho(\vec{r})$. **B)** Isosurfaces of $s(\vec{r})$ = 0.6 colored by sign $(\lambda_2)\rho(\vec{r})$.

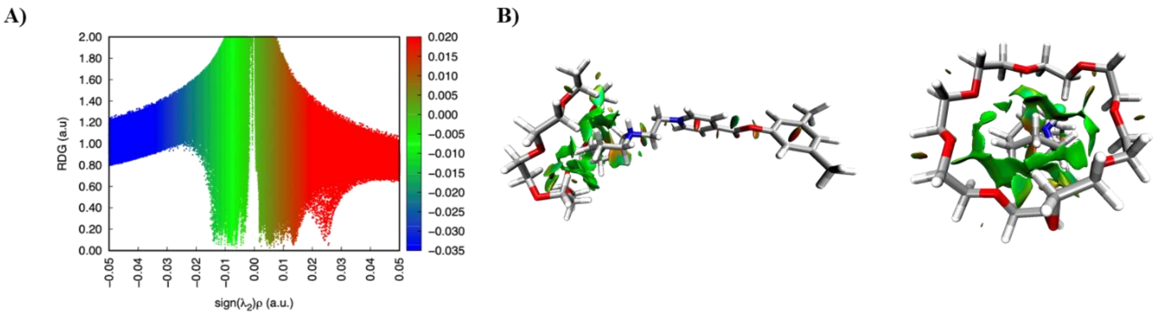


**Figure S15**. Standard NCI index representations of position $\mathbf{P_{127}}$ taken from the 253-point energy profile of the **[1b⊂24C8]**$^{2+}$ complex in **A)** $s(\vec{r})$ plotted against the sign $(\lambda_2)\rho(\vec{r})$. **B)** Isosurfaces of $s(\vec{r})$ = 0.6 colored by sign $(\lambda_2)\rho(\vec{r})$.

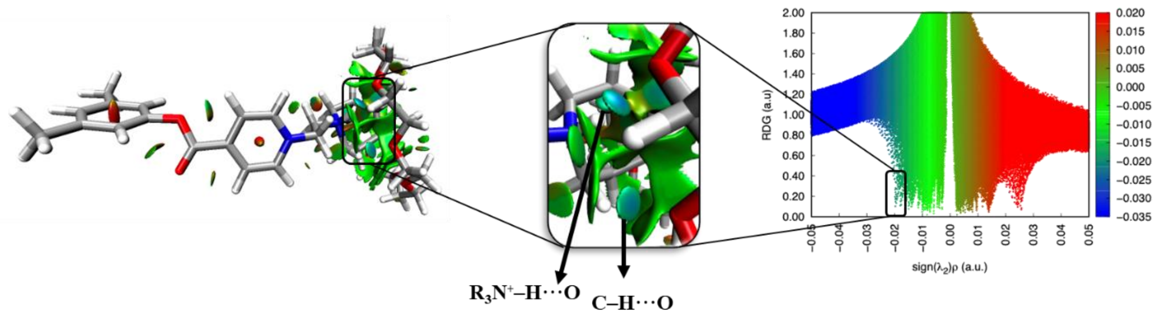

**Figure S16**. Standard NCI index representations of position **P$_{165}$** taken from the 253-point energy profile of the **[1b⊂24C8]$^{2+}$** complex of $s(\vec{r})$ plotted against the sign $(\lambda_2)\rho(\vec{r})$ and isosurfaces of $s(\vec{r})$ = 0.6 colored by sign $(\lambda_2)\rho(\vec{r})$.

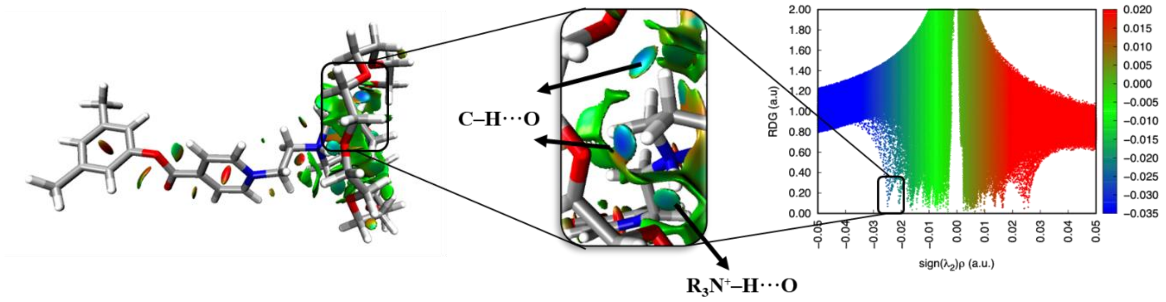


**Figure S17**. Standard NCI index representations of position **P$_{171}$** taken from the 253-point energy profile of the **[1b⊂24C8]$^{2+}$** complex of $s(\vec{r})$ plotted against the sign $(\lambda_2)\rho(\vec{r})$ and isosurfaces of $s(\vec{r})$ = 0.6 colored by sign $(\lambda_2)\rho(\vec{r})$.

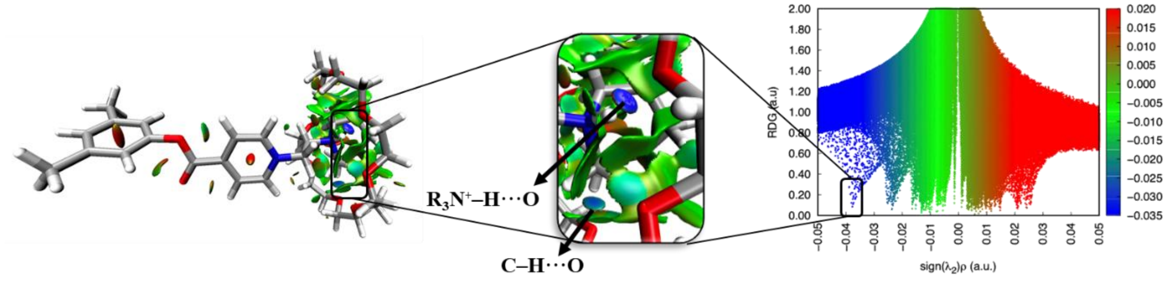


**Figure S18**. Standard NCI index representations of position **P$_{197}$** taken from the 253-point energy profile of the **[1b⊂24C8]$^{2+}$** complex of $s(\vec{r})$ plotted against the sign $(\lambda_2)\rho(\vec{r})$ and isosurfaces of $s(\vec{r})$ = 0.6 colored by sign $(\lambda_2)\rho(\vec{r})$.

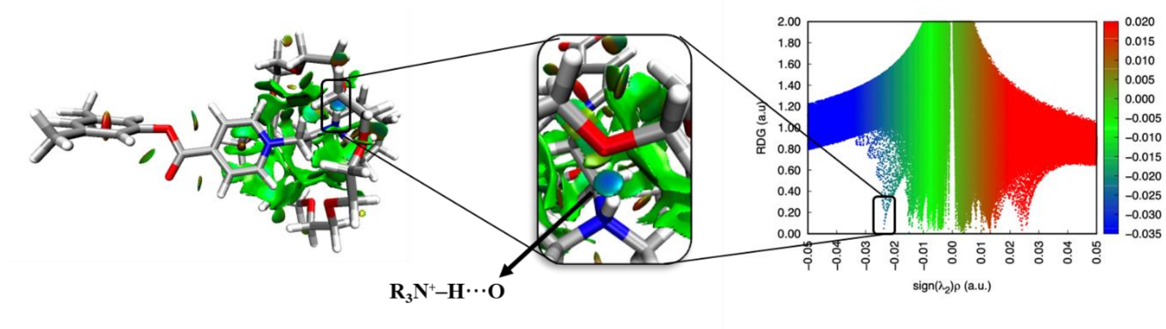

**Figure S19**. Standard NCI index representations of position $\mathbf{P_{228}}$ taken from the 253-point energy profile of the **[1b⊂24C8]$^{2+}$** complex of $s(\vec{r})$ plotted against the sign $(\lambda_2)\rho(\vec{r})$ and isosurfaces of $s(\vec{r})$ = 0.6 colored by sign $(\lambda_2)\rho(\vec{r})$.

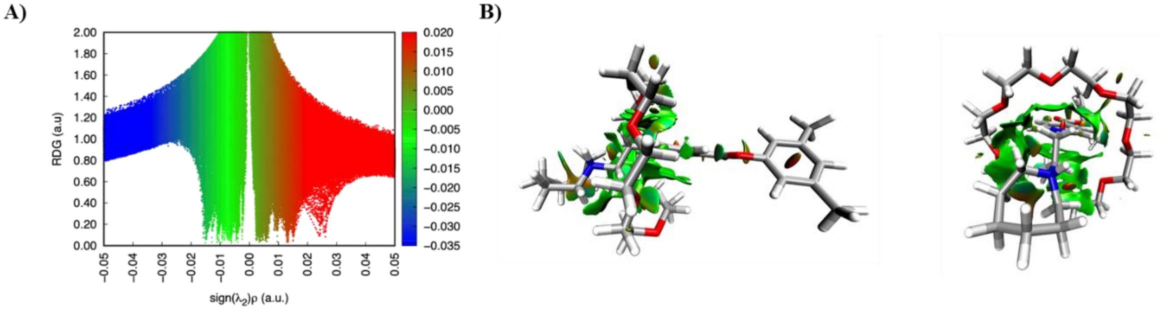


**Figure S20**. Standard NCI index representations of position $\mathbf{P_{253}}$ taken from the 253-point energy profile of the **[1b⊂24C8]$^{2+}$** complex in **A)** $s(\vec{r})$ plotted against the sign $(\lambda_2)\rho(\vec{r})$. **B)** Isosurfaces of $s(\vec{r})$ = 0.6 colored by sign $(\lambda_2)\rho(\vec{r})$.

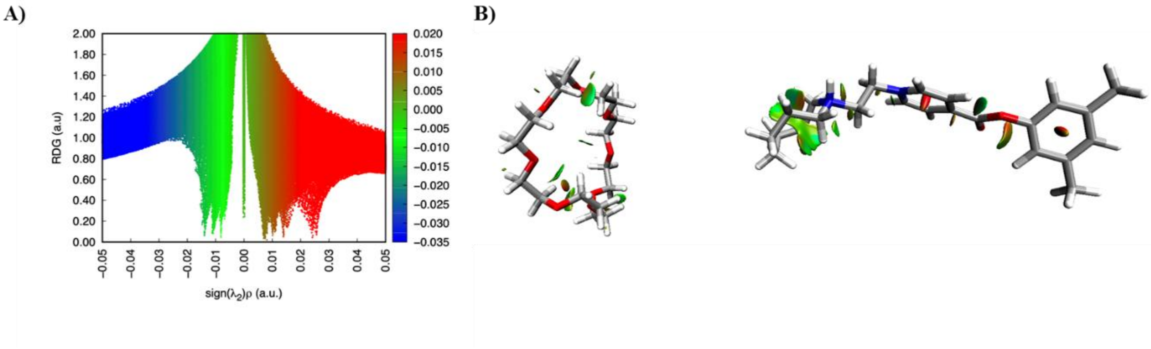


**Figure S21**. Standard NCI index representations of position $\mathbf{P_{1}}$ taken from the 253-point energy profile of the **[1c⊂24C8]$^{2+}$** complex in **A)** $s(\vec{r})$ plotted against the sign $(\lambda_2)\rho(\vec{r})$. **B)** Isosurfaces of $s(\vec{r})$ = 0.6 colored by sign $(\lambda_2)\rho(\vec{r})$.

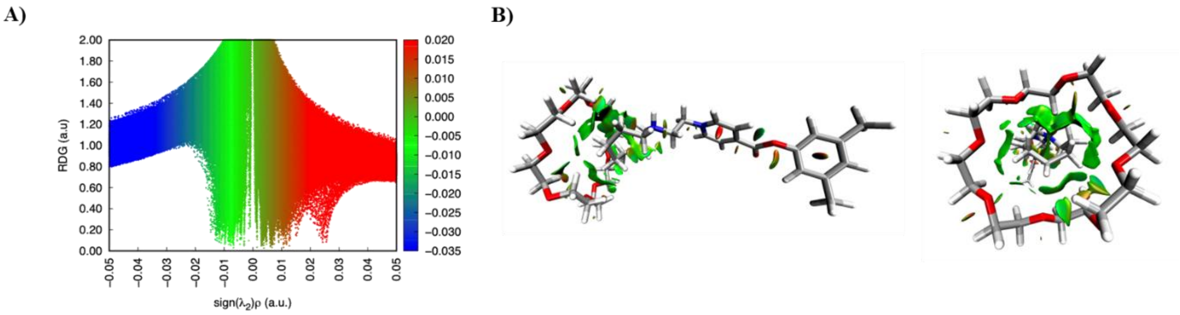

**Figure S22**. Standard NCI index representations of position $\mathbf{P_{127}}$ taken from the 253-point energy profile of the **[1c⊂24C8]$^{2+}$** complex in **A)** $s(\vec{r})$ plotted against the sign $(\lambda_2)\rho(\vec{r})$. **B)** Isosurfaces of $s(\vec{r})$ = 0.6 colored by sign $(\lambda_2)\rho(\vec{r})$.

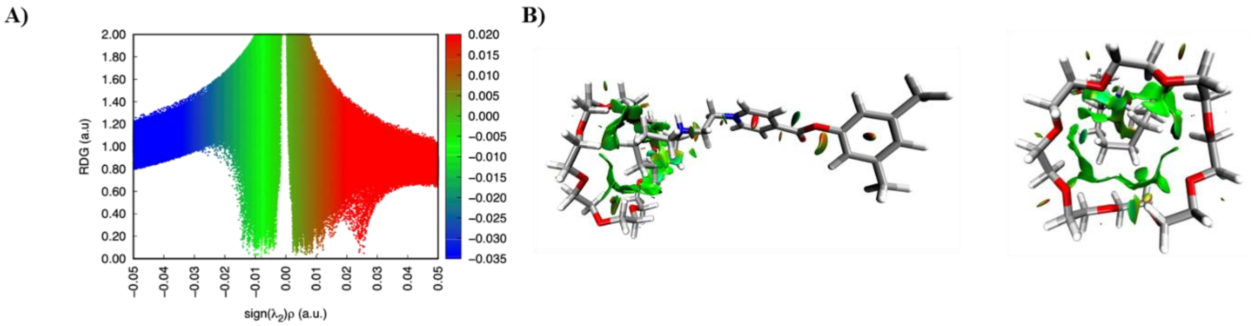


**Figure S23**. Standard NCI index representations of position $\mathbf{P_{150}}$ taken from the 253-point energy profile of the **[1c⊂24C8]$^{2+}$** complex in **A)** $s(\vec{r})$ plotted against the sign $(\lambda_2)\rho(\vec{r})$. **B)** Isosurfaces of $s(\vec{r})$ = 0.6 colored by sign $(\lambda_2)\rho(\vec{r})$.

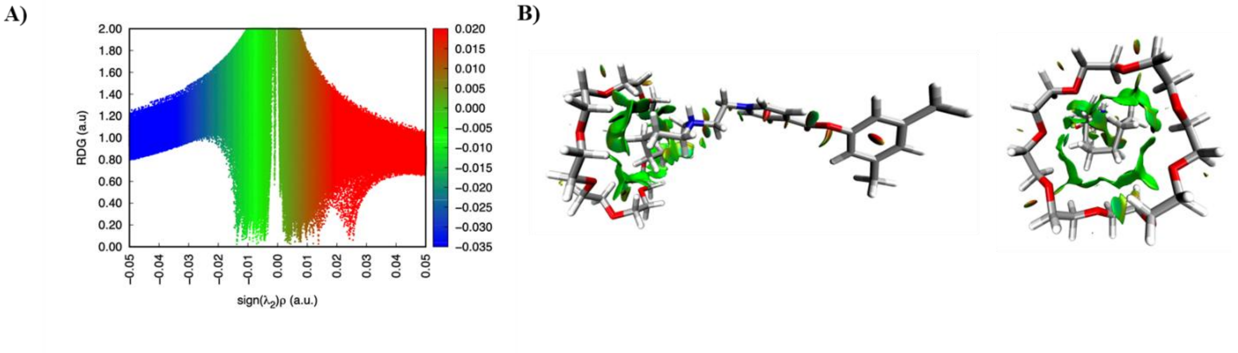


**Figure S24**. Standard NCI index representations of position $\mathbf{P_{169}}$ taken from the 253-point energy profile of the **[1c⊂24C8]$^{2+}$** complex in **A)** $s(\vec{r})$ plotted against the sign $(\lambda_2)\rho(\vec{r})$. **B)** Isosurfaces of $s(\vec{r})$ = 0.6 colored by sign $(\lambda_2)\rho(\vec{r})$.

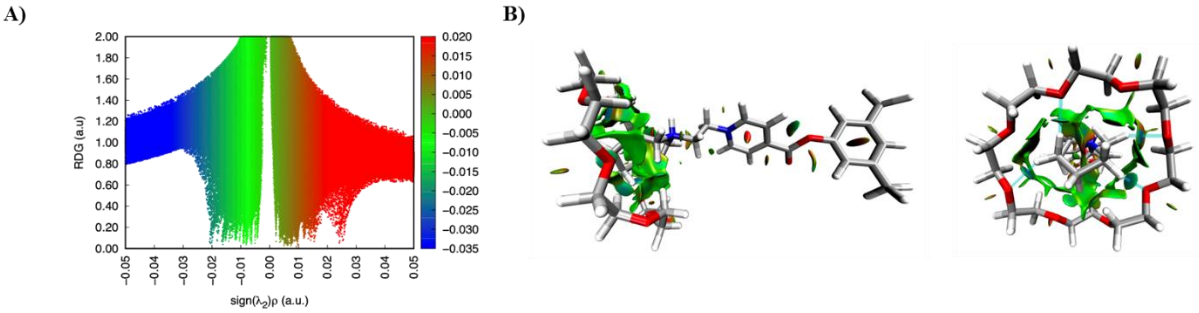

**Figure S25**. Standard NCI index representations of position **P178** taken from the 253-point energy profile of the **[1c⊂24C8]$^{2+}$** complex in **A)** $s(\vec{r})$ plotted against the sign $(\lambda_2)\rho(\vec{r})$. **B)** Isosurfaces of $s(\vec{r})$ = 0.6 colored by sign $(\lambda_2)\rho(\vec{r})$.

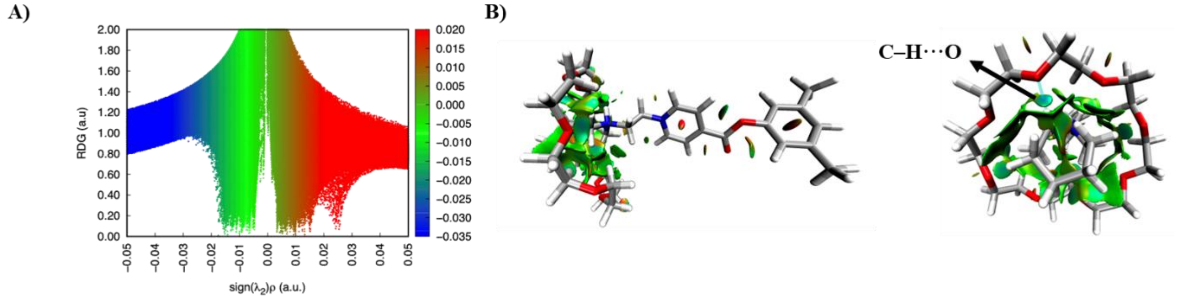


**Figure S26**. Standard NCI index representations of position **P183** taken from the 253-point energy profile of the **[1c⊂24C8]$^{2+}$** complex in **A)** $s(\vec{r})$ plotted against the sign $(\lambda_2)\rho(\vec{r})$. **B)** Isosurfaces of $s(\vec{r})$ = 0.6 colored by sign $(\lambda_2)\rho(\vec{r})$.

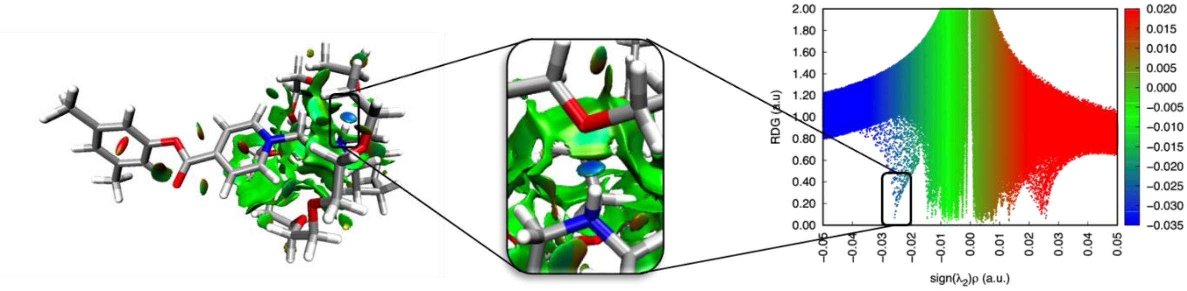


**Figure S27**. Standard NCI index representations of position **P218** taken from the 253-point energy profile of the **[1c⊂24C8]$^{2+}$** complex of $s(\vec{r})$ plotted against the sign $(\lambda_2)\rho(\vec{r})$ and isosurfaces of $s(\vec{r})$ = 0.6 colored by sign $(\lambda_2)\rho(\vec{r})$.

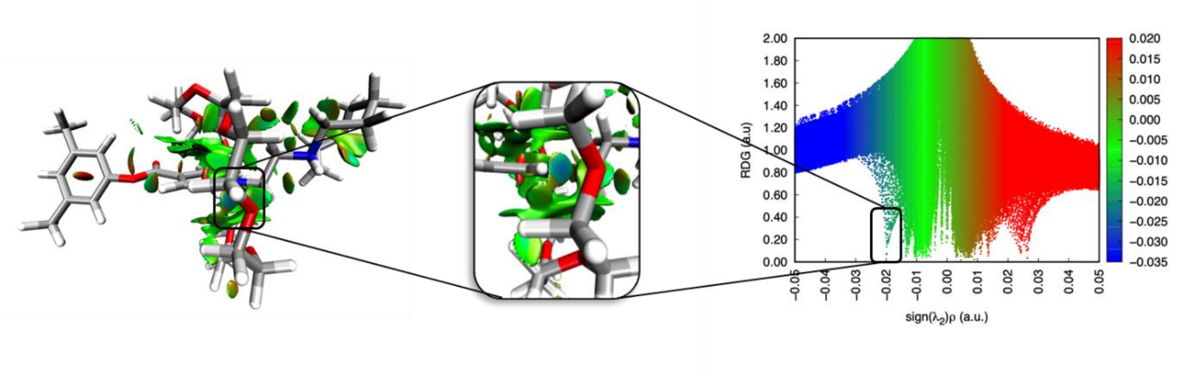

**Figure S28**. Standard NCI index representations of position $\mathbf{P_{253}}$ taken from the 253-point energy profile of the **[1c⊂24C8]**$^{2+}$ complex of $s(\vec{r})$ plotted against the sign $(\lambda_2)\rho(\vec{r})$ and isosurfaces of $s(\vec{r})$ = 0.6 colored by sign $(\lambda_2)\rho(\vec{r})$.

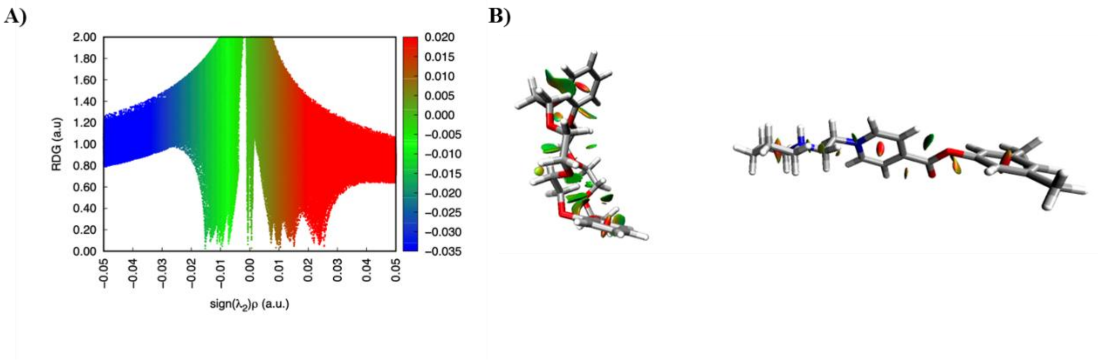


**Figure S29**. Standard NCI index representations of position $\mathbf{P_1}$ taken from the 253-point energy profile of the **[1a⊂DB24C8]**$^{2+}$ complex in **A)** $s(\vec{r})$ plotted against the sign $(\lambda_2)\rho(\vec{r})$. **B)** Isosurfaces of $s(\vec{r})$ = 0.6 colored by sign $(\lambda_2)\rho(\vec{r})$.

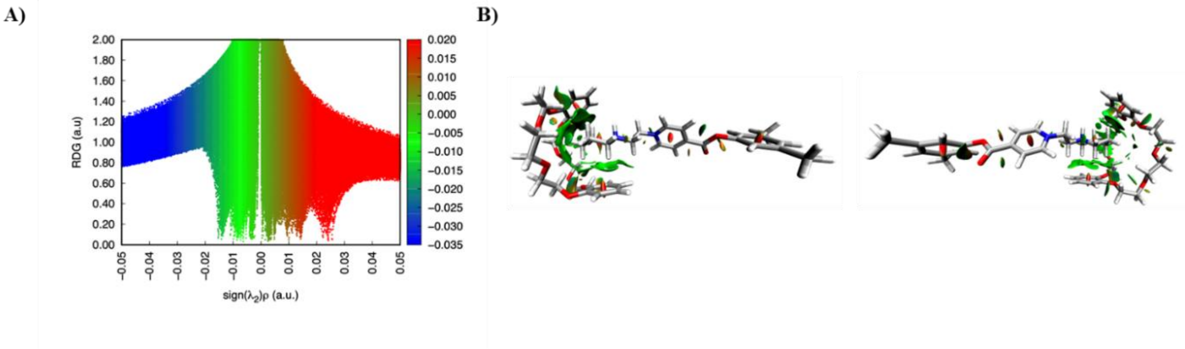


**Figure S30**. Standard NCI index representations of position $\mathbf{P_{127}}$ taken from the 253-point energy profile of the **[1a⊂DB24C8]**$^{2+}$ complex in **A)** $s(\vec{r})$ plotted against the sign $(\lambda_2)\rho(\vec{r})$. **B)** Isosurfaces of $s(\vec{r})$ = 0.6 colored by sign $(\lambda_2)\rho(\vec{r})$.

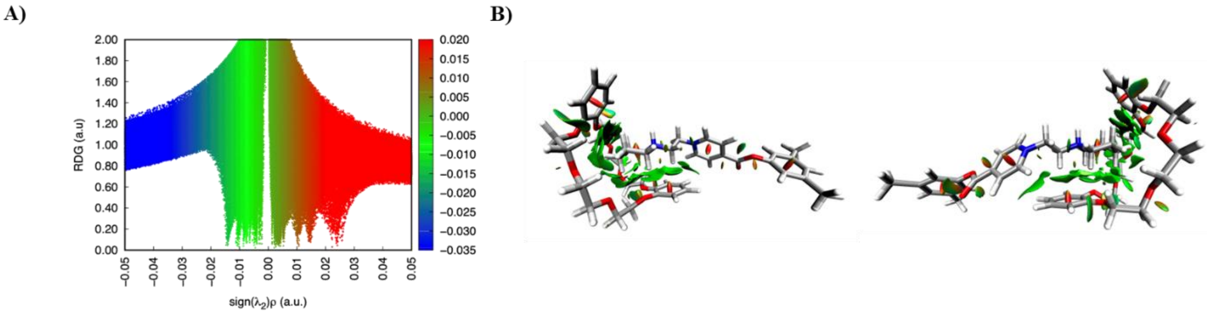

**Figure S31**. Standard NCI index representations of position **P₁₅₁** taken from the 253-point energy profile of the **[1a⊂DB24C8]$^{2+}$** complex in **A)** $s(\vec{r})$ plotted against the sign $(\lambda_2)\rho(\vec{r})$. **B)** Isosurfaces of $s(\vec{r})$ = 0.6 colored by sign $(\lambda_2)\rho(\vec{r})$.

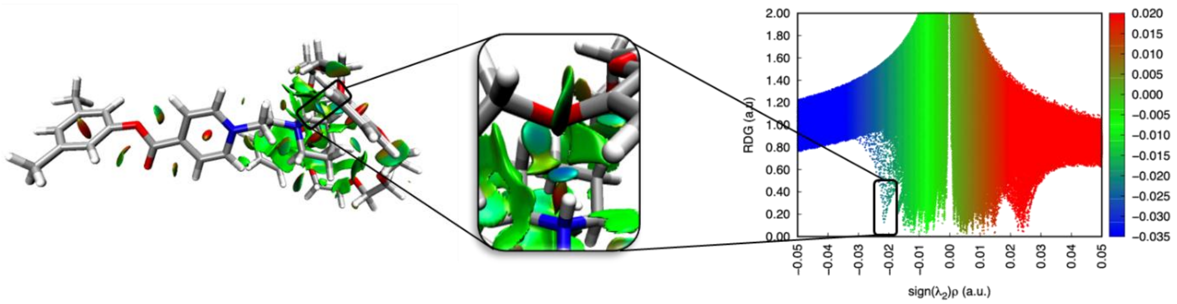


**Figure S32**. Standard NCI index representations of position **P₁₈₁** taken from the 253-point energy profile of the **[1a⊂DB24C8]$^{2+}$** complex of $s(\vec{r})$ plotted against the sign $(\lambda_2)\rho(\vec{r})$ and isosurfaces of $s(\vec{r})$ = 0.6 colored by sign $(\lambda_2)\rho(\vec{r})$.

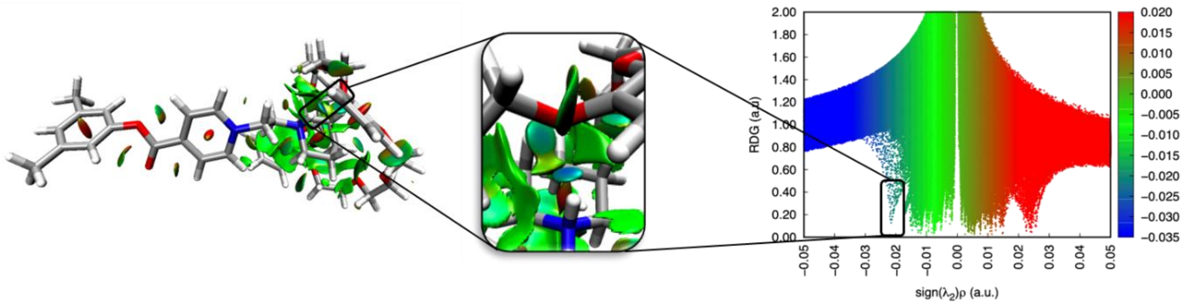


**Figure S33**. Standard NCI index representations of position **P₁₈₆** taken from the 253-point energy profile of the **[1a⊂DB24C8]$^{2+}$** complex of $s(\vec{r})$ plotted against the sign $(\lambda_2)\rho(\vec{r})$ and isosurfaces of $s(\vec{r})$ = 0.6 colored by sign $(\lambda_2)\rho(\vec{r})$.

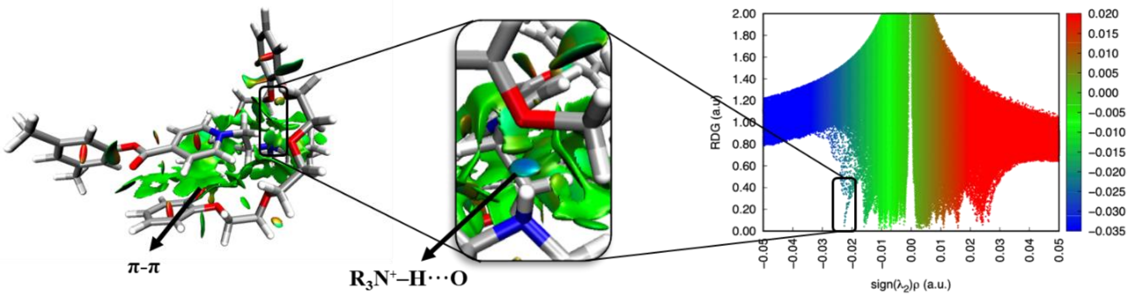

**Figure S34**. Standard NCI index representations of position **$P_{208}$** taken from the 253-point energy profile of the **[1a⊂DB24C8]$^{2+}$** complex of $s(\vec{r})$ plotted against the sign $(\lambda_2)\rho(\vec{r})$ and isosurfaces of $s(\vec{r})$ = 0.6 colored by sign $(\lambda_2)\rho(\vec{r})$.

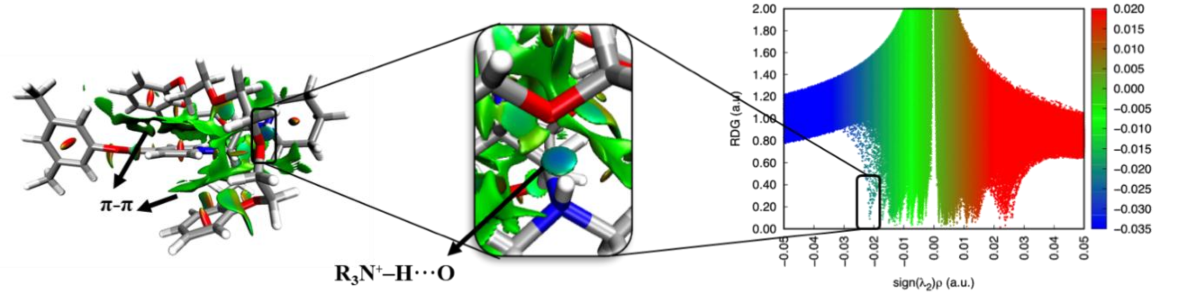


**Figure S35**. Standard NCI index representations of position **$P_{236}$** taken from the 253-point energy profile of the **[1a⊂DB24C8]$^{2+}$** complex of $s(\vec{r})$ plotted against the sign $(\lambda_2)\rho(\vec{r})$ and isosurfaces of $s(\vec{r})$ = 0.6 colored by sign $(\lambda_2)\rho(\vec{r})$.

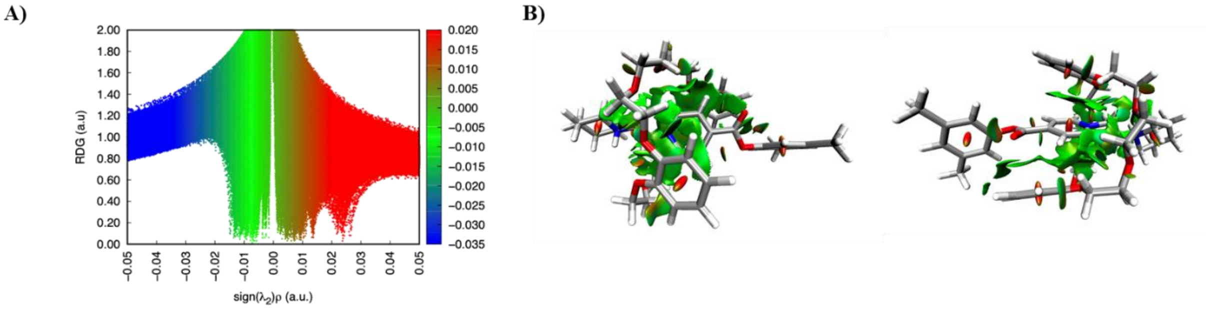


**Figure S36**. Standard NCI index representations of position **$P_{252}$** taken from the 253-point energy profile of the **[1a⊂DB24C8]$^{2+}$** complex in **A)** $s(\vec{r})$ plotted against the sign $(\lambda_2)\rho(\vec{r})$. **B)** Isosurfaces of $s(\vec{r})$ = 0.6 colored by sign $(\lambda_2)\rho(\vec{r})$.

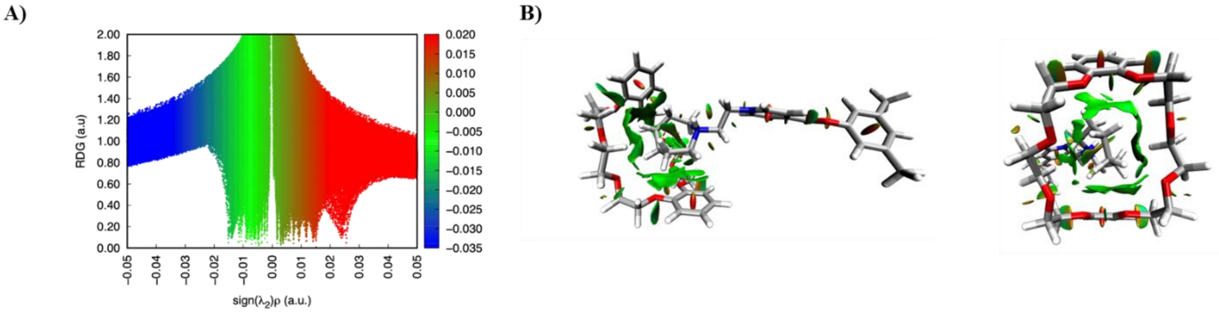

**Figure S37**. Standard NCI index representations of position $\mathbf{P_1}$ taken from the 253-point energy profile of the **[1b⊂DB24C8]$^{2+}$** complex in **A)** $s(\vec{r})$ plotted against the sign $(\lambda_2)\rho(\vec{r})$. **B)** Isosurfaces of $s(\vec{r})$ = 0.6 colored by sign $(\lambda_2)\rho(\vec{r})$.

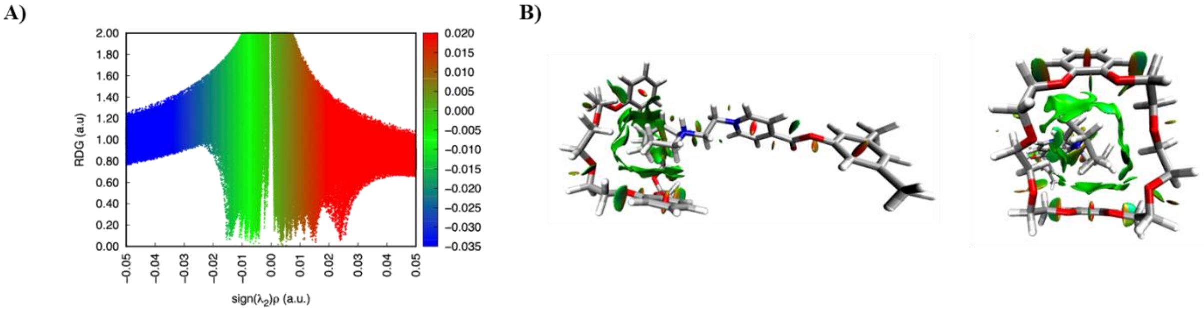


**Figure S38**. Standard NCI index representations of position $\mathbf{P_{138}}$ taken from the 253-point energy profile of the **[1b⊂DB24C8]$^{2+}$** complex in **A)** $s(\vec{r})$ plotted against the sign $(\lambda_2)\rho(\vec{r})$. **B)** Isosurfaces of $s(\vec{r})$ = 0.6 colored by sign $(\lambda_2)\rho(\vec{r})$.

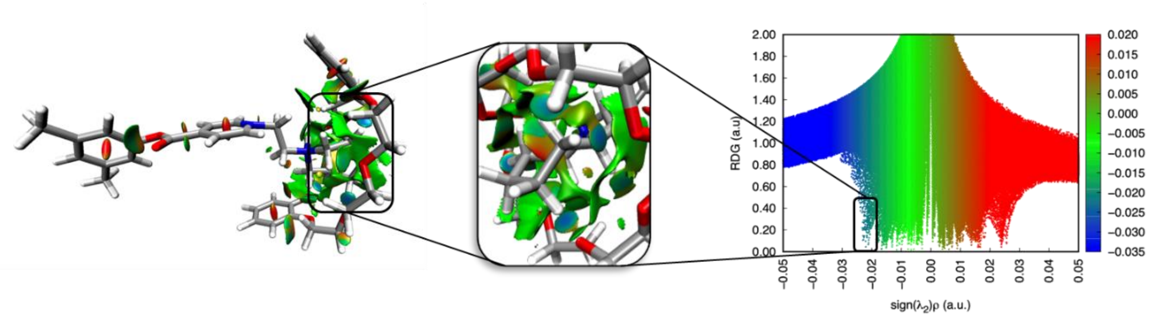


**Figure S39**. Standard NCI index representations of position $\mathbf{P_{176}}$ taken from the 253-point energy profile of the **[1b⊂DB24C8]$^{2+}$** complex of $s(\vec{r})$ plotted against the sign $(\lambda_2)\rho(\vec{r})$ and isosurfaces of $s(\vec{r})$ = 0.6 colored by sign $(\lambda_2)\rho(\vec{r})$.

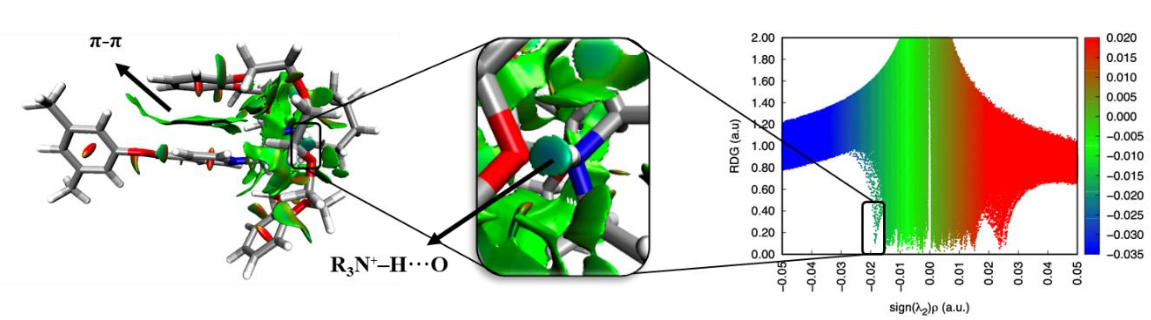

**Figure S40**. Standard NCI index representations of position **$P_{210}$** taken from the 253-point energy profile of the **[1b⊂DB24C8]$^{2+}$** complex of $s(\vec{r})$ plotted against the sign $(\lambda_2)\rho(\vec{r})$ and isosurfaces of $s(\vec{r})$ = 0.6 colored by sign $(\lambda_2)\rho(\vec{r})$.

A) B)

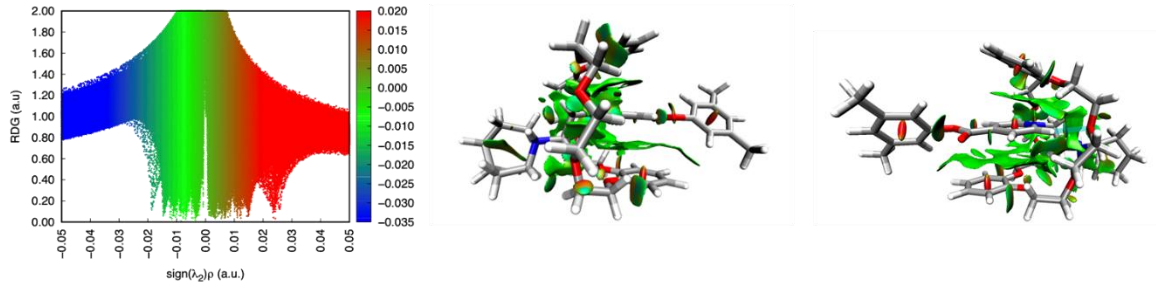


**Figure S41**. Standard NCI index representations of position **$P_{253}$** taken from the 253-point energy profile of the **[1b⊂DB24C8]$^{2+}$** complex in **A)** $s(\vec{r})$ plotted against the sign $(\lambda_2)\rho(\vec{r})$. **B)** Isosurfaces of $s(\vec{r})$ = 0.6 colored by sign $(\lambda_2)\rho(\vec{r})$.

## 4. Plot of the energy and centers of mass distance (CMD)

A) B)

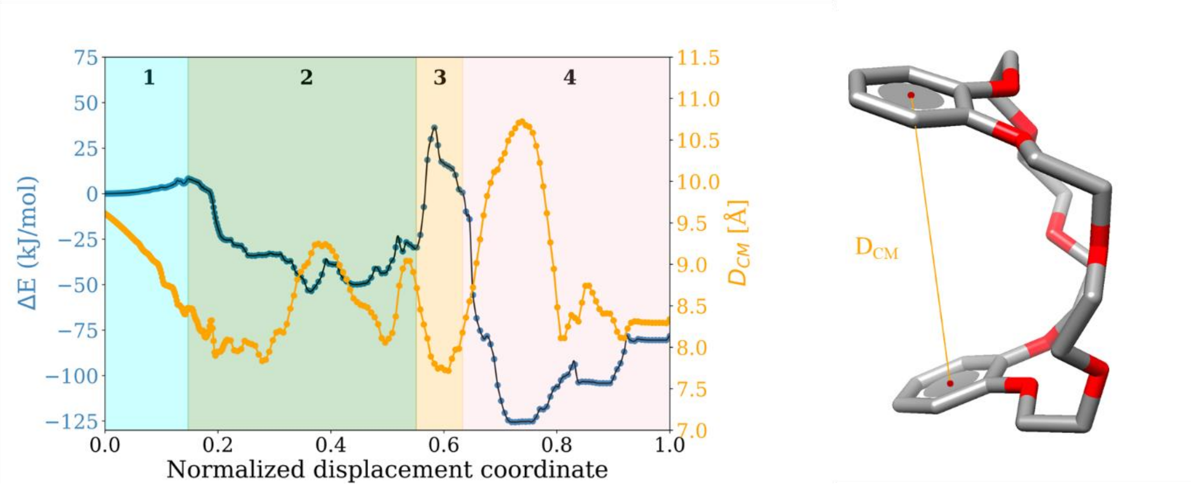


**Figure S42**. Curve (or profile) of energy and centers of mass distance (CMD) of the phenyls of **DB24C8** against the normalized displacement coordinate of **A) [1a⊂DB24C8]$^{2+}$** complex and **B)** representative structures of the CMD in **DB24C8**.

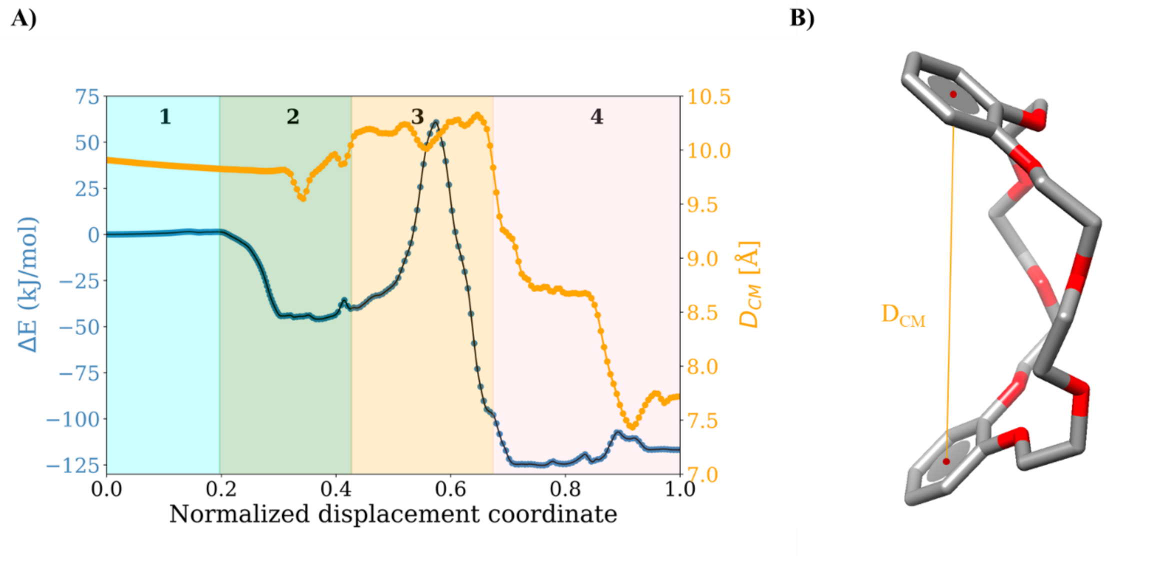


**Figure S43**. Curve (or profile) of energy and centers of mass distance (CMD) of the phenyls of **DB24C8** against the normalized displacement coordinate of **A) [1b⊂DB24C8]$^{2+}$** complex and **B)** representative structures of the CMD in **DB24C8**.

## 5. Conformation of rotaxane

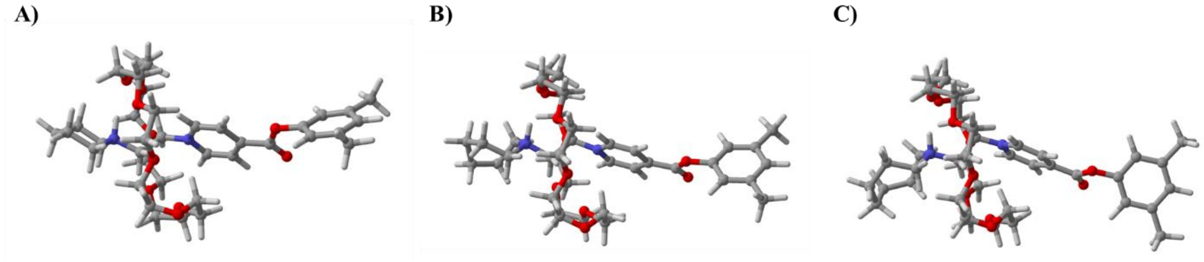


**Figure S44**. S-type conformation of 24C8 in complexes **A) [1a⊂24C8]$^{2+}$**, **B) [1b⊂24C8]$^{2+}$** and **C) [1c⊂24C8]$^{2+}$**.

## 6. References


1 D. Fang, J.-P. Piquemal, S. Liu and G. A. Cisneros, *Theor. Chem. Acc.*, 2014, **133**, 1484.

2 S. Liu, *J. Chem. Phys.*, 2007, **126**, 244103.

3 Y. Huang, A.-G. Zhong, Q. Yang and S. Liu, *J. Chem. Phys.*, 2011, **134**, 84103.

4 J. M. del Campo, J. L. Gázquez, R. J. Alvarez-Mendez and A. Vela, *Int. J. Quantum Chem.*, 2012, **112**, 3594–3598.

5 E. R. Johnson, S. Keinan, P. Mori-Sánchez, J. Contreras-García, A. J. Cohen and W. Yang, *J. Am. Chem. Soc.*, 2010, **132**, 6498–6506.

6 C. Lefebvre, G. Rubez, H. Khartabil, J.-C. Boisson, J. Contreras-García and E. Hénon, *Phys. Chem. Chem. Phys.*, 2017, **19**, 17928–17936.

7 T. Lu and F. Chen, *J. Comput. Chem.*, 2012, **33**, 580–592.
